# Alterations to longitudinal muscle morphology and mechanical function following immobilization and recovery in female ovariectomized and intact rats

**DOI:** 10.1113/EP093152

**Published:** 2026-07-11

**Authors:** Alexandra Q. Kirkup, Amelia Rilling, Alexander M. Zero, Geoffrey A. Power

**Affiliations:** ^1^ Department of Human Health Sciences, College of Biological Sciences University of Guelph Guelph Ontario Canada

**Keywords:** menopause, muscle architecture, oestrogen, power, recovery, sarcomerogenesis

## Abstract

Serial sarcomere number (SSN) is reduced following immobilization of a muscle in a shortened position in male rodents thereby impairing muscle contractile function. However, in female rodents the effects of oestrogen on SSN and mechanical changes following immobilization and re‐ambulation are unknown. Here we investigated the effects of oestrogen deficiency (i.e., ovariectomized; OVX model) on SSN loss, recovery and contractile function following hindlimb immobilization and 4 weeks of voluntary ambulation. In female Sprague–Dawley rats [(Intact; high oestrogen; *n* = 24; mass = 290.6 g ± 25.1 g), (OVX; low oestrogen; *n* = 24; mass = 317.8 g ± 27.3 g)], each left hind‐limb was immobilized in maximal plantar flexion (shortened muscle position) for 14 days. In vivo plantar flexor torque and power were assessed pre‐cast and at 0, 1, 2 and 4 weeks post‐cast. Six rats per group were euthanized at each post‐cast time point for measurements of soleus and medial gastrocnemius SSN. Immediately post‐cast, soleus and medial gastrocnemius SSN and plantar flexor peak torque were reduced similarly in both groups (∼16% and ∼45%, respectively; *P *< 0.05). After 4 weeks of re‐ambulation, intact rats demonstrated full recovery of SSN and torque, whereas OVX rats exhibited persistent deficits (−10% and −30%, respectively; *P *< 0.05). These findings indicate that oestrogen facilitates serial sarcomerogenesis and functional recovery following immobilization in female skeletal muscle.

## Abstract (highlights)

- **What is the central question of this study?**
  Oestrogen deficiency is associated with impaired skeletal muscle recovery: what is its influence on longitudinal muscle remodelling, including serial sarcomere number adaptations and associated mechanical function following immobilization‐induced muscle atrophy and subsequent recovery?
- **What is the main finding and its importance?**
  Using a unilateral hindlimb immobilization model in intact and ovariectomized female rats, it was shown that oestrogen deficiency impairs longitudinal muscle remodelling during recovery following immobilization. These architectural deficits were accompanied by persistent impairments in plantar flexor mechanical function, including torque and power production. These findings provide novel evidence linking oestrogen status to longitudinal muscle remodelling and recovery of muscle mechanical function following disuse.

## 1 INTRODUCTION

While the effects of disuse on muscle architecture have been widely studied (Hinks & Power, 2024; Mirzoev, 2020; Narici & Cerretelli, 1998; Williams & Goldspink, 1978), the influence of sex hormones, particularly oestrogen, on these adaptations remains poorly understood. Oestrogen (i.e., 17β‐oestradiol), interacts with skeletal muscle through specific oestrogen receptors (ERα, ERβ, plasma ER) expressed in muscle fibres and satellite cells to modulate muscle protein turnover and structural remodelling (Collins et al., 2019; Heldrig et al., 2006; Seko et al., 2020). Oestrogen appears to support muscle regeneration and recovery through anti‐apoptotic, anti‐inflammatory and antioxidant mechanisms (Bombardier et al., 2013; Lee et al., 2007; Paroo et al., 2002; Pellegrino et al., 2022; Rogers et al., 2010; Tiidus, 2003; Vasconsuelo et al., 2010). Thus, oestrogen deficiency is associated with muscle atrophy and weakness, with ovariectomized (OVX) female rats exhibiting up to 10% and 18% reductions in muscle cross‐sectional area (CSA) and strength, respectively, as compared with intact female rats (Kitajima & Ono, 2016).

Mechanical unloading, such as hindlimb suspension or immobilization, rapidly induces muscle atrophy, especially in postural muscles like the soleus (Greenhaff, 2006; Hinks & Power, 2024, 2025; Sitnick et al., 2006; Warren et al., 2004). Importantly, females may have some protection from muscle atrophy during mechanical unloading. During disuse, ovariectomy exacerbated soleus muscle atrophy and blunted recovery, where OVX rats had a 60% greater loss in muscle wet weight than intact controls following 10 days of hindlimb suspension (McClung et al., 2006). While some studies have reported no significant differences in muscle atrophy between intact and OVX animals during unloading (Brown et al., 2005; Fisher et al., 1998), oestrogen replacement consistently accelerates post‐unloading recovery of muscle mass and fibre size (Brown et al., 2005; McClung et al., 2006; Sitnick et al., 2006). In addition to preserving muscle mass, oestrogen may also contribute to recovery of muscle contractile function. Female rats demonstrated greater grip strength restoration and improved fatigue resistance compared to males during 7 days of reloading following hindlimb suspension (Issertine et al., 2024; Mortreux et al., 2021). Collectively, oestrogen may offer some protection of muscle form and function during and following periods of mechanical unloading.

Although changes in muscle mass and CSA typically describe radial remodelling, the recovery of contractile performance also depends on longitudinal remodelling, specifically the addition or loss of sarcomeres in series (Hinks et al., 2022; Hornberger, 2025; Rilling et al., 2025). Serial sarcomerogenesis occurs in response to sustained changes in muscle length from stretch, immobilization or exercise training (Hinks & Power, 2024; Hornberger, 2025; Rilling et al., 2025; Sitnick et al., 2006; Vidal et al., 2020; Williams & Goldspink, 1978). In human male adults, even a short 10‐day bed‐rest protocol shortens biceps femoris fascicles by ∼3%, demonstrating that serial remodelling also occurs in adult skeletal muscle (Sarto et al., 2021; Yeo, 2025). Work in animals further supports this such that in young male rats, serial sarcomere number (SSN) decreased by ∼22% after 2 weeks of casting the soleus in a shortened position but recovered fully during 3–4 weeks of re‐ambulation (Hinks & Power, 2024). Similarly, after 3 weeks of immobilization of the plantar flexors in a shortened position there was a ∼23% SSN loss in male Wistar rats (Coutinho et al., 2004; Gomes et al., 2004). Conversely, immobilizing a muscle in a lengthened position results in the addition of sarcomeres in series, with Hinks et al. (2023) reporting an ∼6% increase and Williams & Goldspink (1978) reporting an ∼9% increase in soleus SSN. Furthermore, intermittent passive stretching during immobilization in a shortened position may also limit SSN loss if applied at a high frequency (Coutinho et al., 2004; Gomes et al., 2004; Vidal et al., 2020; Williams, 1988, 1990; Ywazaki et al., 2016). Altogether, SSN appears highly responsive to chronic or repetitive changes in muscle length.

While it is clear the mechanical loading environment modulates SSN adaptation, the mechanisms underlying this remodelling, including potential hormonal influences such as oestrogen, remain poorly understood. Stretching interventions in female rats with differing oestrogen levels provide early evidence for SSN modulation; for example, Ywazaki et al. (2016) observed ∼8% greater SSN in intact compared to OVX female rats following 6 weeks of twice‐weekly static stretching (10 × 1 min static stretches with 45 s rest intervals for 6 weeks). More recently, Vidal et al. (2020) compared intact and OVX female rats under the same stretch protocol (10 × 1 min static stretches with 45 s rest intervals for 3 weeks) and found a non‐statistically significant (∼15%) SSN increase in the soleus of intact rats that was absent in OVX rats, a result likely influenced by limited statistical power due to small sample sizes. Together, this work provides some evidence that ovarian hormones, particularly oestrogen, may facilitate longitudinal muscle remodelling; however, these studies did not include direct mechanical measurements, and further controlled studies using rigorous disuse models are needed to clarify this relationship.

The purpose of the current study was to investigate: (1) oestrogen‐mediated differences in the loss of soleus SSN and plantar flexor mechanical function after 2 weeks of casting in a shortened muscle length; and (2) oestrogen‐mediated differences in the recovery of soleus SSN and plantar flexor mechanical function throughout a 4‐week period of voluntary ambulation following cast removal. We hypothesized that OVX rats would exhibit a greater magnitude of SSN loss compared to intact females immediately following immobilization, and experience slower SSN recovery, greater mechanical impairments, and blunted longitudinal muscle growth due to the absence of oestrogen's protective role against disuse atrophy and in modulating muscle recovery following unloading.

## 2 METHODS

### 2.1 Ethical approval

All protocols were approved by the University of Guelph's Animal Care Committee (AUP no. 4905) and followed guidelines from the Canadian Council on Animal Care.

Forty‐eight intact female adult (*n* = 24) (14–20 weeks; 290.6 ± 25.1 g) and ovariectomized (OVX) female adult (*n* = 24) (14‐20 weeks; 317.8 ± 27.3 g) Sprague–Dawley rats were obtained (Charles River Laboratories, Laval, QC, Canada).

**FIGURE 1:**
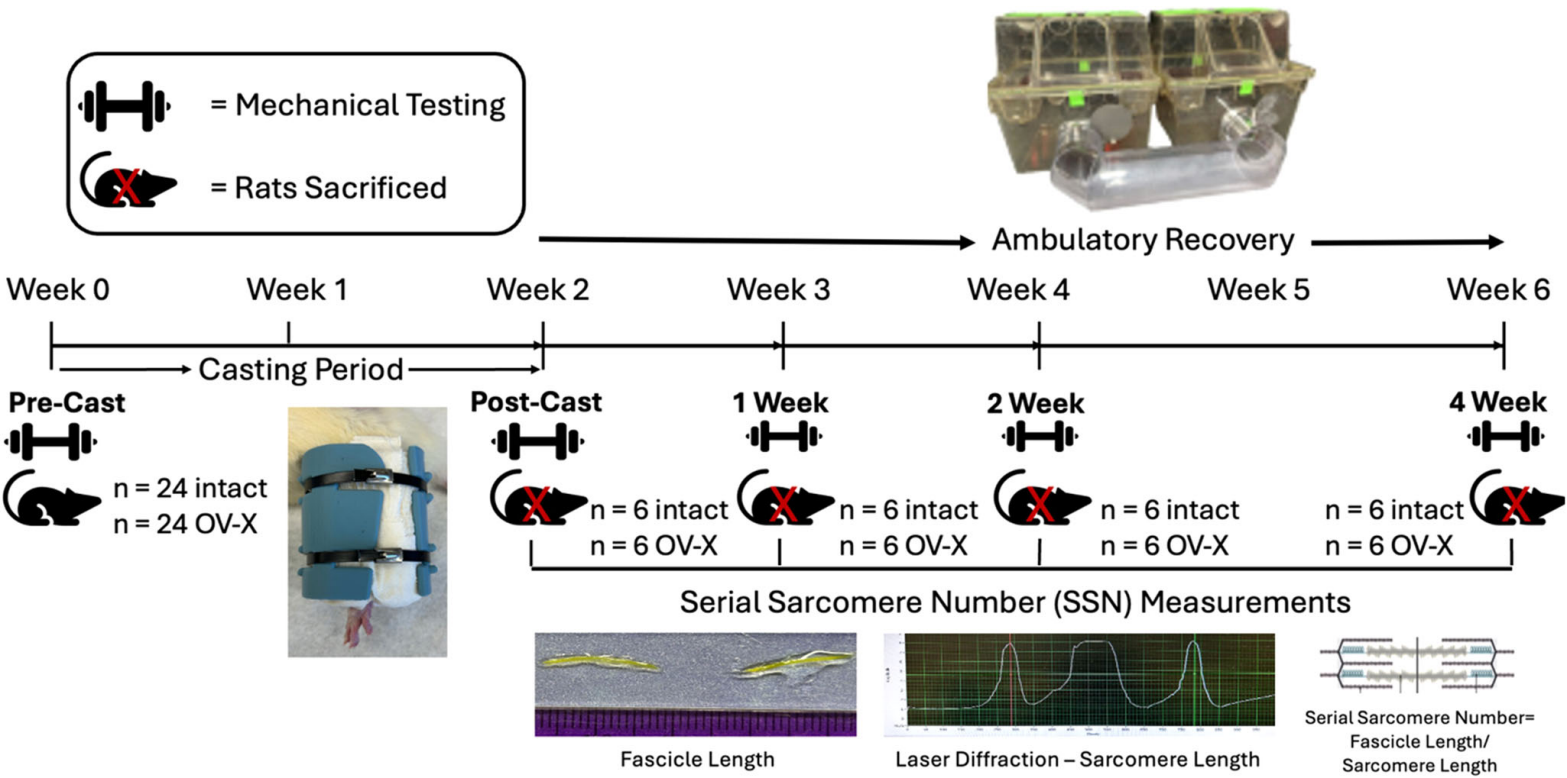
Experimental time line outlining the casting and recovery protocol. Female rats (*n* = 48; 24 intact and 24 ovariectomized (OVX)) underwent in vivo mechanical testing of the plantar flexors prior to immobilization (Pre‐Cast). All animals were unilaterally immobilized with a custom cast on the left hindlimb for 2 weeks. Casts were replaced after 1 week to ensure integrity. Subsets of rats (*n* = 6 per group) were sacrificed at four time points: immediately post‐cast, and at 1‐, 2‐ and 4‐weeks post‐cast. Prior to sacrifice, all rats underwent the same in vivo mechanical testing of the plantar flexors that was performed prior to casting (Figure 2). Muscles were harvested to assess architectural properties including serial sarcomere number (SSN), average sarcomere length (SL) and fascicle length (FL). Average SL was determined using laser diffraction of isolated fascicles, where diffraction band spacing was used to calculate mean sarcomere length across the fascicle. Fascicle length was measured using digital image analysis, and SSN was calculated by dividing FL by average SL. Ambulatory recovery occurred in the home cage.

Rats were housed at 23°C in groups of two or three, maintained on a 12‐h light–dark cycle, and given ad libitum access to a Teklad global 18% protein rodent diet (Envigo, Huntington, UK) and room‐temperature water. All rats underwent mechanical testing (see below) prior to casting to establish an individual baseline for mechanical muscle function. Mechanical testing measurements were obtained at five time points: pre‐cast, 0 week/immediately post‐cast, 1‐week recovery, 2‐week recovery and 4‐week recovery (Figure 1). Six intact female and six OVX female rats were sacrificed immediately following cast removal (week 0/post‐cast time point), six intact female and six OVX female rats were sacrificed 1 week following cast removal, six intact female and six OVX female rats were sacrificed 2 weeks following cast removal, and six intact female and six OVX female rats were sacrificed 4 weeks following cast removal (Figure 1). Following sacrifice, both the right (control) and left (casted) hindlimbs were fixed in formalin for subsequent determination of soleus and gastrocnemius SSN. In accordance with previous studies (Heslinga & Huijing, 1993; Hinks et al., 2023; Williams & Goldspink, 1978), for architectural measures, the left leg was used as the experimental leg; meanwhile, the right leg acted as an internal control.

### 2.2 Ovariectomy procedure

Ovariectomized female rats were obtained from Charles River Laboratories, where all surgical procedures were performed by trained staff under the oversight of their Institutional Animal Care and Use Committee (IACUC) prior to arrival at our facility. Animals were ≥21 days old at the time of surgery and received standard pre‐ and postoperative care according to Charles River guidelines. For the surgical procedure, a small dorsal incision was made parallel to the midline, and the skin was retracted to expose the underlying abdominal wall. The abdominal cavity was entered via a blunt puncture, and the ovary and associated fat pad were exteriorized. The fallopian tube was cauterized, the ovary was removed, and the remaining fat pad and tissue were returned to the abdominal cavity. The procedure was then repeated on the contralateral side. The skin incision was closed using wound clips, which were removed 7–10 days following surgery. Postoperatively, animals were held for a minimum of 24 h, with most shipments occurring within 7 days of surgery. Rats were ovariectomized for approximately 21 days prior to undergoing any experimental procedures. The intact rats did not undergo a sham procedure. Although circulating oestradiol levels were not directly measured in the present study, previous work using a comparable OVX rat model (Chang et al., 2019) demonstrated circulating oestradiol concentrations of <5 pg/mL within 3 weeks following OVX surgery, remaining substantially lower than intact females through 6 weeks post‐OVX.

### 2.3 Unilateral limb immobilization

The left hindlimb of each rat was immobilized in full plantar flexion for 2 weeks using gauze padding, self‐adherent vet wrap, and a custom 3D‐printed brace and splint (Supporting information, Figure S1). To monitor for swelling, the toes were left exposed throughout the casting period (Aoki et al., 2009; Hinks & Power, 2024, 2025). Casts were inspected daily and repaired or replaced as necessary. As the rats were allowed unrestricted cage ambulation while casted, the intervention induced muscular atrophy of only the immobilized muscles without full mechanical unloading, in contrast to models such as hindlimb suspension (Issertine et al., 2024; Rosa‐Caldwell et al., 2023; Warren et al., 2004).

### 2.4 Mechanical testing

**FIGURE 2:**
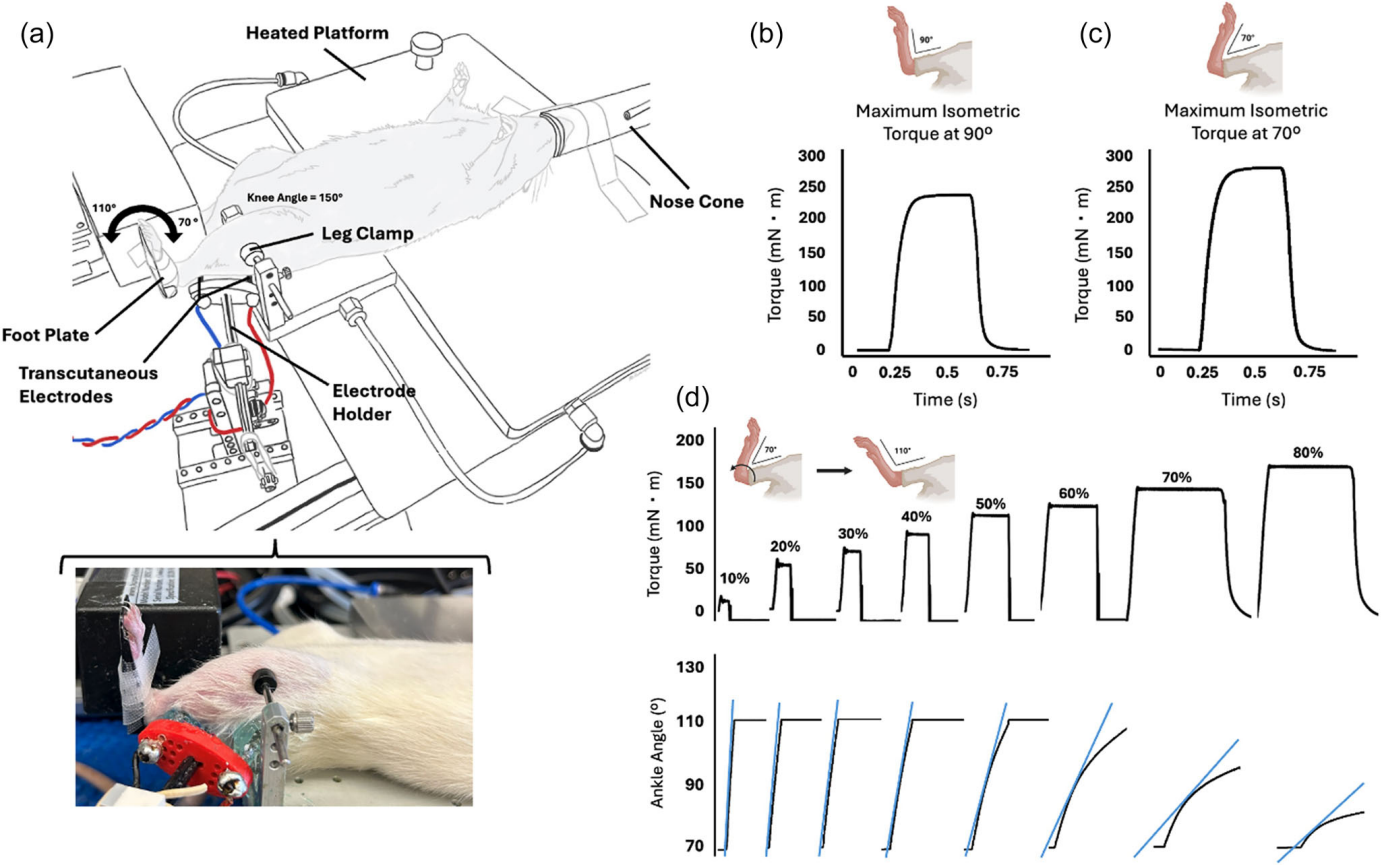
Experimental set‐up for in vivo assessment of rat plantar flexor mechanics. (a) Schematic representation of the experimental apparatus. Anaesthetized rats were placed prone on a heated platform with the left leg clamped at the knee (~150°) and the foot strapped to a pedal connected to a torque transducer. Percutaneous stimulation electrodes positioned over the tibial nerve delivered supramaximal square‐wave pulses. (b, c) Representative isometric torque traces evoked at fixed ankle angles of 90° (b; short muscle length) and 70° (c; long muscle length). (d) Torque and ankle angle traces from dynamic contractions at increasing isotonic loads (10–80% of maximum) during passive plantar flexor shortening from 70° to 110°. Blue lines on angle traces represent fitted slopes used to calculate shortening velocity.

During mechanical testing, a 701C High‐Powered, Bi‐Phase Stimulator (Aurora Scientific, Aurora, ON, Canada) was used to transcutaneously evoke muscle contractions via a custom‐built electrode holder (Figure 2). Pilot testing confirmed that this set‐up yielded consistent maximum isometric tetanic torque values across repeated sessions and produced plantar flexion torque magnitudes comparable to those reported in previous literature employing direct nerve stimulation (Hinks & Power, 2024, 2025; Hinks et al., 2025; Padilla et al., 2021). Torque, joint angle and stimulation trigger signals were sampled at 1000 Hz using a 605A Dynamic Muscle Data Acquisition and Analysis System (Aurora Scientific), with live torque tracings displayed during all mechanical data collection sessions. All baseline mechanical measurements were obtained prior to casting when rats were the same age and before assignment into recovery groups. Thus, minor differences in pre‐cast values observed across recovery time points reflect independent cohorts and random biological variation between groups rather than longitudinal changes in the same animals over time.

Rats were anaesthetized using inhaled isoflurane administered until a surgical plane of anaesthesia was reached (typically within 3–5 min) and maintained throughout all procedures until completion. Rats were then positioned in a supine position on a heated platform maintained at 37°C. After complete removal of fur from the left hindlimb using a hand‐held electric razor and hair removal cream, the foot was secured to a force transducer and length controller (Aurora Scientific) via a custom foot pedal using adhesive tape, with the knee immobilized at a fixed angle of ∼150°. The ankle joint angle was defined as the angle between the tibia and the sole of the foot, where 180° represented full plantar flexion. Each session began by determining the optimal stimulation current at an ankle angle of 90°, using a 100 Hz train frequency, 0.1 ms pulse duration and 500 ms train duration. This current was used throughout the remainder of testing and was verified to evoke maximal plantar flexor activation without any antagonist (dorsiflexor) recruitment. This was confirmed by applying a 10‐mA increase in current; a subsequent drop in torque output indicated spillover to the antagonists and confirmed that the prior current reflected near‐maximal plantar flexor stimulation with no co‐contraction. To characterize the torque–velocity–power relationship of the plantar flexor muscles, isometric torque measurements were obtained at ankle angles of 70°, against a fixed resistive load. Loads were set at 10%, 20%, 30%, 40%, 50%, 60%, 70% and 80% of the peak isometric torque previously measured at 70°. The sequence of load conditions was randomized using a custom randomization script to minimize systematic order effects. Two minutes of rest were given between contractions to minimize the development of muscle fatigue. Active torque was calculated by subtracting baseline passive torque from the peak total torque recorded during contraction (Chen et al., 2020; Hinks & Power, 2024; Hinks et al., 2022). Passive torque was assessed at both 70° and 90° ankle angles following a 5‐s rest period. Angular velocity was calculated as the average slope of the position–time trace during the shortening phase of each contraction. Power output was computed as the product of torque and angular velocity for each load. The relationship between torque and angular velocity was fitted to a rectangular hyperbolic curve, allowing estimation of key contractile parameters: maximum shortening velocity at zero load (*V*_max_), peak power, and the torque and velocity values at which peak power occurred.

The above measures were calculated using the Hill equation (Hill, 1938; Seow, 2013):

$$\left(F+a\right)\left(v+b\right)=\left(F_{\max}+a\right)b$$

### 2.5 Muscle harvesting and sarcomere number determination

Following their final mechanical testing session, rats were euthanized by isoflurane anaesthesia followed by CO_2_ asphyxiation. Both the left (CAST) and right (CON) hindlimbs were amputated, skinned, and all overlying muscles surrounding the soleus and gastrocnemius were carefully removed. The limb was then fixed in 10% phosphate‐buffered formalin with the ankle joint held at 90°. After 1–2 weeks of fixation, the soleus and medial gastrocnemius muscles were carefully dissected to separate them from the lower leg, weighed and re‐submerged in formalin until subsequent analysis. The soleus and medial gastrocnemius were both selected for architectural analyses because both muscles were maintained in a chronically shortened position throughout immobilization. Although plantar flexor mechanical output reflects contributions from multiple muscles, testing was performed with the knee flexed, mechanically disadvantaging the biarticular gastrocnemius and increasing the relative contribution of the monoarticular soleus during plantar flexor torque production. Additionally, the medial gastrocnemius was selected for architectural analyses rather than the whole gastrocnemius muscle (lateral and medial gastrocnemius combined) due to its more consistent fascicle organization and reduced variability during fascicle dissection and isolation for serial sarcomere number measurements (Hinks et al., 2023). To begin SSN estimations, muscles were rinsed in phosphate‐buffered saline and digested in 30% nitric acid for 5–6 h to remove connective tissue and facilitate the separation of individual muscle fascicles (Butterfield et al., 2005; Butterfield & Herzog, 2005; Hinks et al., 2022; Hinks & Power, 2024). From each soleus and medial gastrocnemius muscle, two fascicles were isolated from the proximal, middle and distal thirds (*n* = 6 fascicles per muscle). This regional sampling strategy was based on prior work demonstrating minimal regional variability in SSN within the rat soleus (Hinks et al., 2022, 2023; Hinks & Power, 2024, 2025). Individual fascicles were mounted on glass microslides (VWR International, Radnor, PA, USA) and photographed using a tripod‐mounted digital camera aligned perpendicular to the slide. Fascicle lengths (FL) were measured in ImageJ (version 1.53f, NIH, Bethesda, MD, USA), with scale calibration based on an in‐plane ruler. Sarcomere length (SL) was measured at six different locations along each fascicle, from proximal to distal (total of *n* = 36 SL measurements per muscle), using laser diffraction (5 mW diode laser, 635 nm wavelength; Coherent, Santa Clara, CA, USA) and a custom LabVIEW program (Version 2011, National Instruments, Austin, TX, USA) (Lieber et al., 1984). Each laser diffraction measurement, assuming a beam diameter of ∼1 mm, represented the average length of thousands of sarcomeres. For each fascicle, the six SL values were averaged to yield a mean SL, and the standard deviation (SL SD) was recorded as an index of sarcomere length non‐uniformity.

Serial sarcomere number for each fascicle was calculated as fascicle length/average sarcomere length. SSN and FL were averaged across the six fascicles per muscle for group‐level comparisons. The number of fascicles and SL measurements per muscle (*n* = 6 fascicles × 6 SLs = 36 total) is consistent with established methodological standards in the field (Butterfield et al., 2005; Butterfield & Herzog, 2005; Chen et al., 2020; Hinks et al., 2022, 2023; Hinks & Power, 2024, 2025).

### 2.6 Estimation of physiological cross‐sectional area

To assess changes in muscle architecture and contractile tissue arranged in parallel, the physiological cross‐sectional area (PCSA, in cm^2^) was calculated using the following equation (Ward & Lieber, 2005):

PCSA=musclemass/(muscledensity×normalizedFL)

PCSA calculations were performed for both control and casted soleus and medial gastrocnemius muscles at four post‐intervention time points: immediately following casting (0 weeks), after 1 week of recovery, after 2 weeks of recovery and after 4 weeks of recovery. Muscle mass was taken as the wet weight of the dissected soleus and medial gastrocnemius muscles. Muscle density was assumed to be 1.112 g/cm^3^, consistent with previously reported values (Ward & Lieber, 2005). Normalized fascicle length was determined for each sample by correcting for sarcomere length using the equation (Ward & Lieber, 2005):

$$\mathrm{Normalized}\;\mathrm{FL}\;=\;\mathrm{FL}\;\frac{SL_{o}}{\;\mathrm{measured}\;\mathrm{SL}}$$

An optimal sarcomere length (SL_o_) of 2.7 µm was assumed for rat muscle under resting conditions, based on established literature values (Chen et al., 2020; Zuurbier et al., 1995). This approach allowed us to estimate functional muscle cross‐sectional area under the assumption of constant density and architecture, providing insight into the extent of muscular remodelling in response to disuse and recovery.

### 2.7 Calculation of normalized torque

Owing to our set‐up, mechanical testing was performed on the left plantar flexors. Pre‐cast torque measurements were obtained from the limb that was subsequently subjected to immobilization. PCSA could only be determined following euthanasia and tissue collection. Therefore, pre‐cast torque values were normalized using PCSA from the contralateral control limb collected at the terminal time point for each cohort, whereas post‐cast torque values were normalized using PCSA from the corresponding casted limb. Normalized torque was included to provide an estimate of force production relative to muscle size. The formula used to calculate normalized torque was:

$$\mathrm{Normalized}\;\mathrm{Torque}\;=\frac{\mathrm{Active}\;\mathrm{torque}}{\mathrm{Combined}\;\mathrm{PCSA}\;}$$

### 2.8 Statistical analysis

All statistical analyses were performed in SPSS Statistics Premium version 28 (IBM Corp., Armonk, NY, USA). For each variable of interest (e.g., mechanical [peak torque, passive tension, power, velocity] and architectural measures [FL, SL, SSN, muscle wet weight, muscle PCSA]), a univariate three‐way analysis of variance (ANOVA) was conducted using Group (intact vs. OVX), Cast (pre/CON vs. post/CAST), and Time (post‐cast, 1 week, 2 weeks, 4 weeks) as fixed factors. The dependent variable was the raw measurement at each time point, and all factors were treated as independent. To assess recovery trajectories independent of absolute baseline differences, a second analysis was performed on percentage difference values, calculated individually for each rat as the proportional change from pre‐cast (for mechanical measures) or the proportional deficit between CON and CAST legs (for architectural measures). These percentage difference values were analysed using a two‐way ANOVA (Group × Time) to determine whether the magnitude of the immobilization‐induced impairment and its recovery differed between intact and OVX rats across the recovery period. For all ANOVAs, partial eta squared (η^2^) values were obtained to evaluate effect size. When a significant main effect of Time or a significant interaction (e.g., Group × Time, Group × Cast, Time × Cast, Group × Time × Cast) was identified, *post hoc* comparisons were performed and *post hoc P*‐values were adjusted using the Holm–Šidák correction to control the family‐wise error rate. These adjusted *P*‐values were used to determine statistical significance for comparisons within or between groups at individual time points. Statistical significance was set at α ≤ 0.05 for all analyses and effect size is presented as η^2^.

## 3 RESULTS

### 3.1 Mechanical adaptations

#### 3.1.1 Active peak torque at a 90 degree ankle angle

**FIGURE 3:**
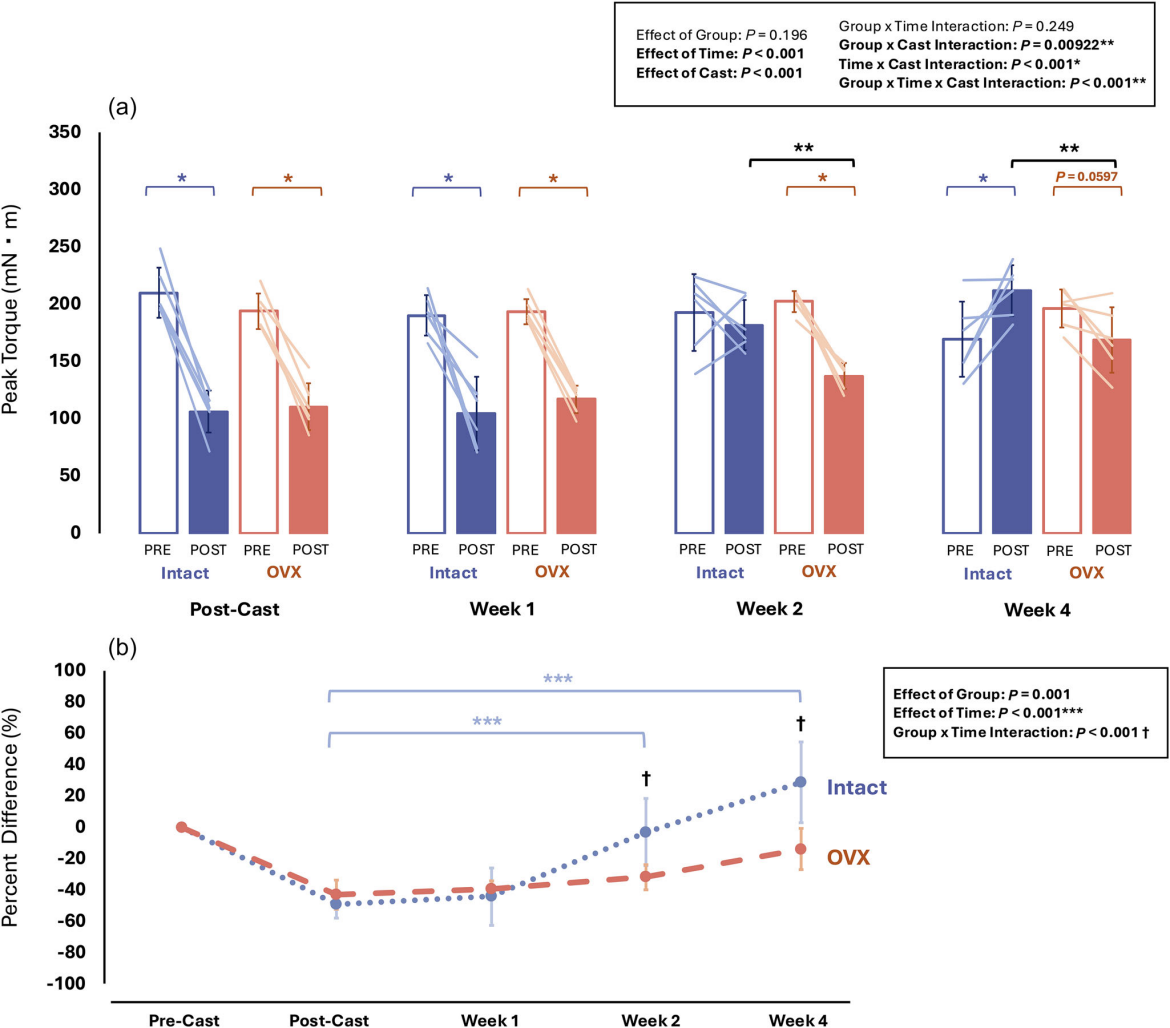
Differences in active plantar flexor torque at a 90° ankle angle in intact (*n* = 24) and ovariectomized (OVX; *n* = 24) adult female rats across recovery time points following unilateral hindlimb immobilization in a shortened position. Data are displayed as means ± standard deviation. (a) Absolute pre‐ and post‐cast torque values for intact and OVX rats at post‐cast, 1 week, 2 weeks and 4 weeks of recovery (*n* = 6/group per time point). Horizontal lines represent the decrease in pre‐ to post‐cast torque values for individual rats. Statistical comparisons were performed using a three‐way ANOVA (Group × Time × Cast). *Significant pre–post difference within a group at that time point (*P < *0.05). **Significant difference between intact and OVX post‐cast values at that time point (*P < *0.05). (b) Percentage difference in torque from pre‐cast to post‐cast for each rat across the same recovery time points. Statistical comparisons were performed using a two‐way ANOVA (Group × Time) on percentage difference values. †Significant difference between intact and OVX groups percentage difference at that time point (*P < *0.05). ***Significant difference from the post‐cast time point when pre‐ and post‐cast values are combined; purple bars represent the intact group (*P < *0.05).

Absolute active peak torque at a 90° ankle angle (Figure 3a) showed significant effects of Time (*P <* 0.001; η^2^ = 0.364) and Cast (*P <* 0.001; η^2^ = 626), as well as significant Group × Cast (*P =* 0.00922; η^2^ = 0.082), Time × Cast (*P <* 0.001; η^2^ = 0.500), and Group × Time × Cast interactions (*P <* 0.001; η^2^ = 0.195). Percentage difference between pre‐ and post‐cast torque (Figure 3b) showed significant effects of Time (*P <* 0.001; η^2^ = 0.701) and Group (*P <* 0.001; η^2^ = 0.227), and a significant Group × Time interaction (*P <* 0.001; η^2^ = 0.365), indicating that the recovery of cast‐induced torque loss differed between intact and OVX groups. Immediately following cast removal, intact and OVX groups experienced similarly large decreases in torque (−49% and −43% from pre‐cast, respectively, *P <* 0.001). However, the recovery trajectory differed between groups, such that in the intact group, torque increased steadily across the recovery period until by week 2 it no longer differed from pre‐cast values (−3% from pre‐cast, *P = *0.374), ultimately surpassing baseline by week 4 (+28% from pre‐cast, *P <* 0.001). In contrast, the OVX group remained significantly impaired at week 2 (–30% from pre‐cast, *P <* 0.001); however, by week 4 there was no significant difference from pre‐cast (−14%, *P* = 0.0597).

#### 3.1.2 Active peak torque at a 90 degree ankle angle normalized to PCSA

**FIGURE 4:**
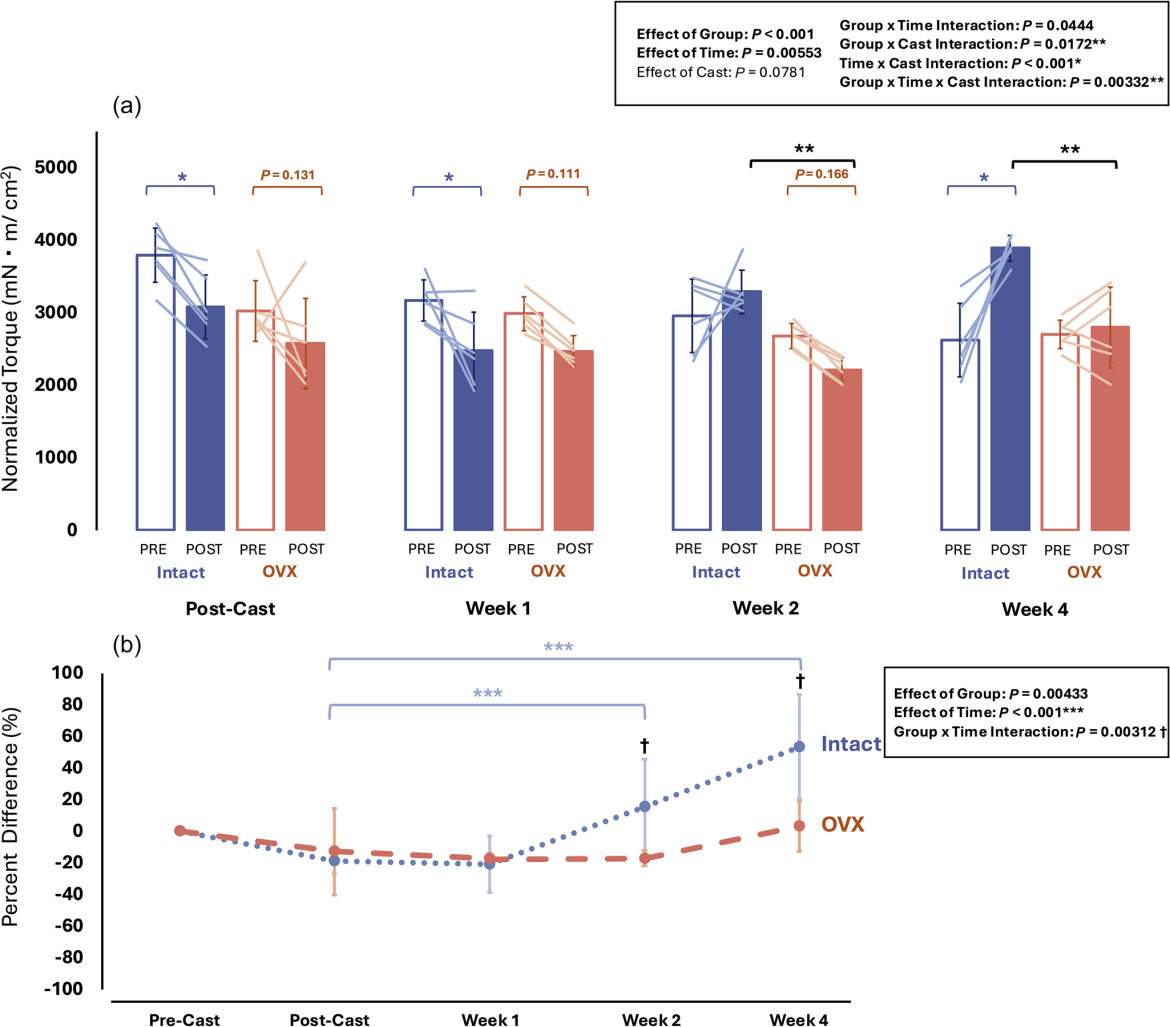
Differences in active plantar flexor torque at a 90° ankle angle normalized to physiological cross‐sectional area (PCSA) in intact (*n* = 24) and ovariectomized (OVX; *n* = 24) adult female rats across recovery time points following unilateral hindlimb immobilization in a shortened position. Data are displayed as means ± standard deviation. (a) Absolute pre‐ and post‐cast torque values for intact and OVX rats at post‐cast, 1 week, 2 weeks and 4 weeks of recovery (*n* = 6/group per time point). Horizontal lines represent the decrease in pre‐ to post‐cast torque values for individual rats. Statistical comparisons were performed using a three‐way ANOVA (Group × Time × Cast). *Significant pre–post difference within a group at that time point (*P < *0.05). **Significant difference between intact and OVX post‐cast values at that time point (*P < *0.05). (b) Percentage difference in normalized torque from pre‐cast to post‐cast for each rat across the same recovery time points. Statistical comparisons were performed using a two‐way ANOVA (Group × Time) on percentage difference values. †Significant difference between intact and OVX groups percentage difference at that time point (*P < *0.05). ***Significant difference from the post‐cast time point when pre‐ and post‐cast values are combined; purple bars represent the intact group (*P < *0.05).

Absolute normalized active peak torque at a 90° ankle angle (Figure 4a) showed significant effects of Group (*P <* 0.001; η^2^ = 0.315) and Time (*P =* 0.00553; η^2^ = 0.145), as well as significant Group × Time (*P =* 0.0444; η^2^ = 0.096), Group × Cast (*P =* 0.0172; η^2^ = 0.069), Time × Cast (*P <* 0.001; η^2^ = 0.353), and Group × Time × Cast interactions (*P =* 0.00332; η^2^ = 0.158). Percentage difference between pre‐ and post‐cast normalized torque (Figure 4b) showed significant effects of Time (*P <* 0.001; η^2^ = 0.494) and Group (*P =* 0.00433; η^2^ = 0.191), and a significant Group × Time interaction (*P =* 0.00312; η^2^ = 0.286), indicating that both the magnitude and the recovery of cast‐induced normalized torque loss differed between intact and OVX rats. Immediately following cast removal, intact (−18% from pre‐cast, *P <* 0.001) and OVX (−13% from pre‐cast, *P = *0.131) groups experienced decreases in normalized torque; however, the OVX group did not show a significant difference between pre‐ and post‐cast values at the post‐cast time point. The recovery trajectory also differed between groups such that, in the intact group, normalized torque increased steadily across the recovery period and by week 2 no longer differed from pre‐cast values (+15% from pre‐cast, *P* = 0.141), ultimately surpassing baseline by week 4 (+54% from pre‐cast, *P <* 0.001). In contrast, at week 2, the OVX group exhibited lower values than intact rats, although they no longer differed from pre‐cast levels. By week 4, the OVX group had recovered to pre‐cast values (+3% from pre‐cast, *P *= 0.664), though not significantly surpassing baseline like the intact group.

#### 3.1.3 Active peak torque at a 70 degree ankle angle

**FIGURE 5:**
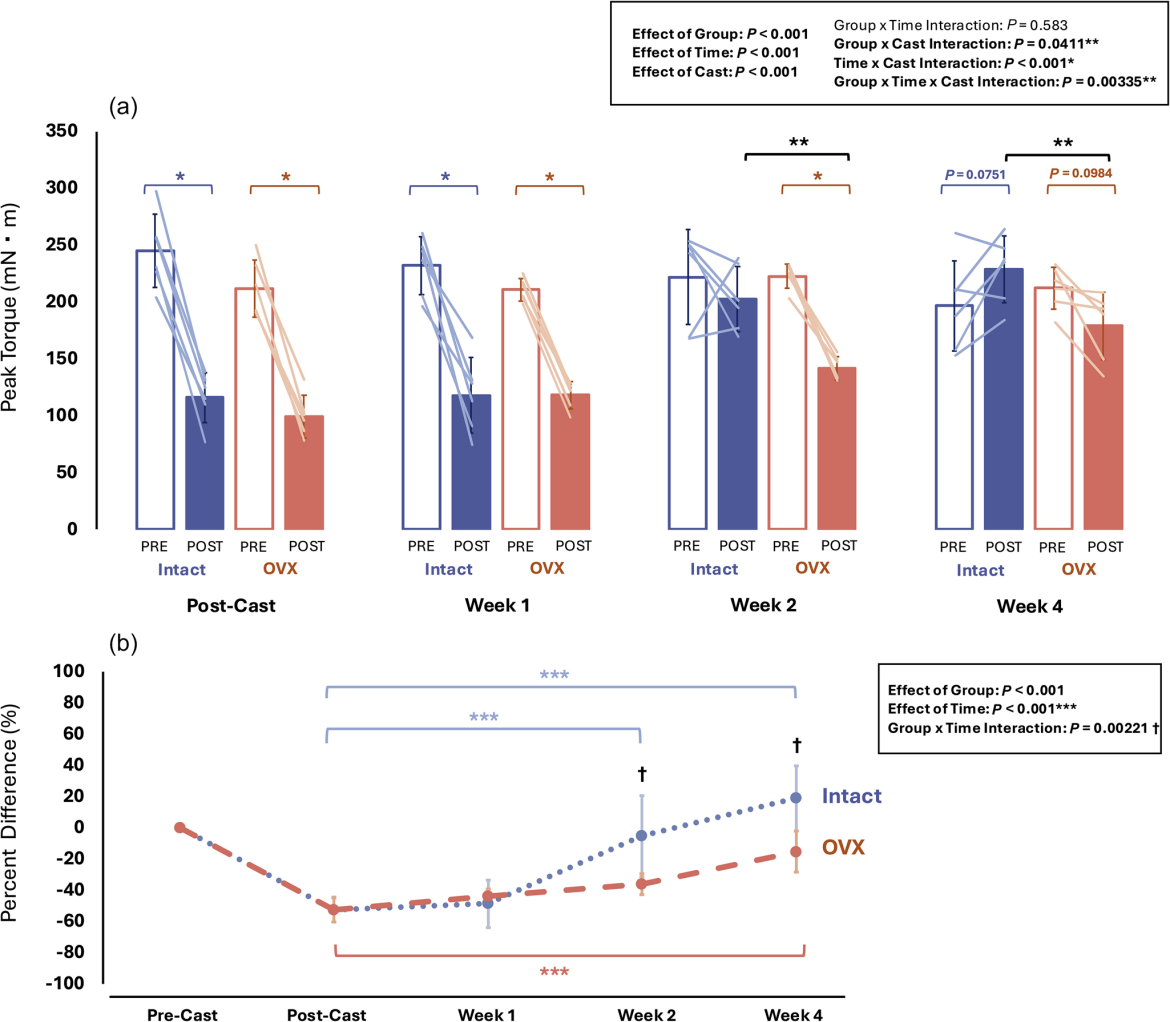
Differences in active plantar flexor torque at a 70° ankle angle in intact (*n* = 24) and ovariectomized (OVX; *n* = 24) adult female rats across recovery time points following unilateral hindlimb immobilization in a shortened position. Data are displayed as means ± standard deviation. (a) Absolute pre‐ and post‐cast torque values for intact and OVX rats at post‐cast, 1 week, 2 weeks and 4 weeks of recovery (*n* = 6/group per time point). Horizontal lines represent the decrease in pre‐ to post‐cast torque values for individual rats. Statistical comparisons were performed using a three‐way ANOVA (Group × Time × Cast). *Significant pre–post difference within a group at that time point (*P < *0.05). **Significant difference between intact and OVX post‐cast values at that time point (*P < *0.05). (b) Percentage difference in torque from pre‐cast to post‐cast for each rat across the same recovery time points. Statistical comparisons were performed using a two‐way ANOVA (Group × Time) on percentage difference values. †Significant difference between intact and OVX groups percentage difference at that time point (*P < *0.05). ***Significant difference from the post‐cast time point when pre‐ and post‐cast values are combined; purple bars represent intact group and orange bar represents OVX group (*P < *0.05).

Absolute active peak torque at a 70° ankle angle (Figure 5a) showed significant effects of Group (*P <* 0.001; η^2^ = 0.157), Time (*P <* 0.001; η^2^ = 0.312), and Cast (*P <* 0.001; η^2^ = 0.676), as well as significant Group × Cast (*P =* 0.041; η^2^ = 0.0411), Time × Cast (*P <* 0.001; η^2^ = 0.495), and Group × Time × Cast interactions (*P =* 0.00335; η^2^ = 0.156). Percentage difference between pre‐ and post‐cast torque (Figure 5b) showed significant effects of Time (*P <* 0.001; η^2^ = 0.724) and Group (*P <* 0.001; η^2^ = 0.245), and a significant Group × Time interaction (*P =* 0.00221; η^2^ = 0.299), indicating that the recovery of cast‐induced torque loss differed between intact and OVX groups. Immediately following cast removal, intact and OVX groups experienced similarly large decreases in torque (−52% and −53% from pre‐cast, respectively, *P <* 0.001). However, the recovery trajectory differed between groups such that, in the intact group, torque increased steadily across the recovery period and by week 2 no longer differed from pre‐cast values (−5% from pre‐cast, *P *= 0.196), ultimately surpassing baseline by week 4 (+18% from pre‐cast, *P =* 0.0751). In contrast, by week 2 the OVX group tended to exhibit lower values than intact rats, and by week 4 continued to show evidence of a persistent torque deficit, although values were not significantly different from pre‐cast levels (–16% from pre‐cast, *P *= 0.0984).

#### 3.1.4 Active peak torque at a 70 degree ankle angle normalized to PCSA

**FIGURE 6:**
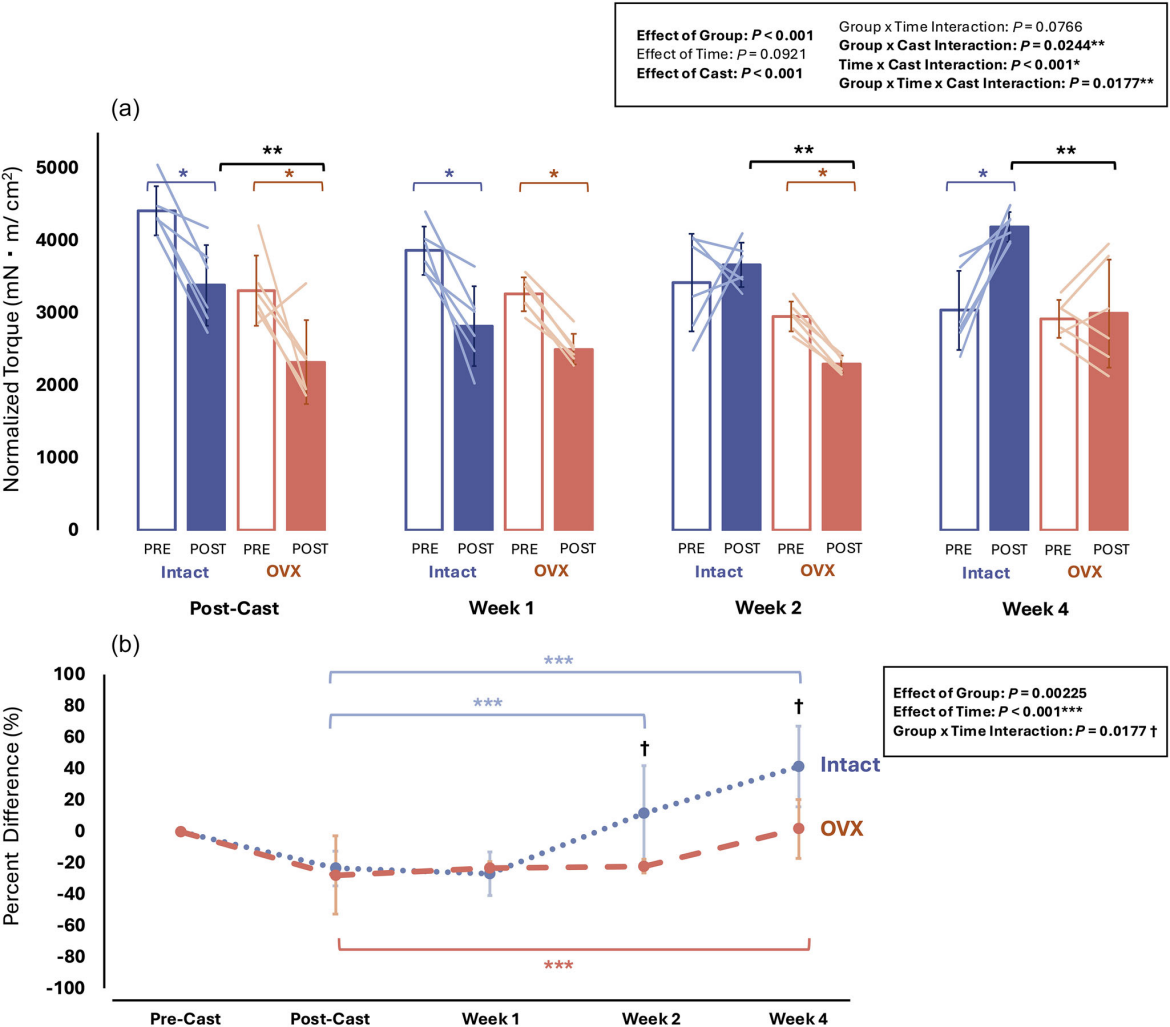
Differences in active plantar flexor torque at a 70° ankle angle normalized to physiological cross‐sectional area (PCSA) in intact (*n* = 24) and ovariectomized (OVX; *n* = 24) adult female rats across recovery time points following unilateral hindlimb immobilization in a shortened position. Data are displayed as means ± standard deviation. (a) Absolute pre‐ and post‐cast torque values for intact and OVX rats at post‐cast, 1 week, 2 weeks and 4 weeks of recovery (*n* = 6/group per time point). Horizontal lines represent the decrease in pre‐ to post‐cast torque values for individual rats. Statistical comparisons were performed using a three‐way ANOVA (Group × Time × Cast). *Significant pre–post difference within a group at that time point (*P < *0.05). **Significant difference between intact and OVX post‐cast values at that time point (*P < *0.05). (b) Percentage difference in normalized torque from pre‐cast to post‐cast for each rat across the same recovery time points. Statistical comparisons were performed using a two‐way ANOVA (Group × Time) on percentage difference values. †Significant difference between intact and OVX groups percentage difference at that time point (*P < *0.05). ***Significant difference from the post‐cast time point when pre‐ and post‐cast values are combined; purple bars represent intact group and orange bar represents OVX group (*P < *0.05).

Absolute normalized active peak torque at a 70° ankle angle (Figure 6a) showed significant effects of Group (*P <* 0.001; η^2^ = 0.487) and Cast (*P <* 0.001; η^2^ = 0.182), as well as significant Group × Cast (*P =* 0.0244; η^2^ = 0.062), Time × Cast (*P <* 0.001; η^2^ = 0.396), and Group × Time × Cast interactions (*P =* 0.0177; η^2^ = 0.118). Percentage difference between pre‐ and post‐cast torque (Figure 6b) showed significant effects of Time (*P <* 0.001; η^2^ = 0.553) and Group (*P =* 0.00225; η^2^ = 0.225), and a significant Group × Time interaction (*P =* 0.0177; η^2^ = 0.223), indicating that the recovery of cast‐induced torque loss differed between intact and OVX groups. Immediately following casting, intact and OVX groups experienced similarly large decreases in normalized torque (−23% and −27% from pre‐cast, respectively, *P <* 0.001). However, the recovery trajectory differed between groups such that, in the intact group, torque increased steadily across the recovery period and by week 2 no longer differed from pre‐cast values (+11% from pre‐cast, *P *= 0.341), ultimately surpassing baseline by week 4 (+41% from pre‐cast, *P <* 0.001). In contrast, the OVX group remained significantly impaired at week 2 (−22% from pre‐cast, *P =* 0.00411). By week 4, the OVX group had recovered to pre‐cast values (+1% from pre‐cast, *P = *0.774), though values remained lower than the intact group.

#### 3.1.5 Peak power

**FIGURE 7:**
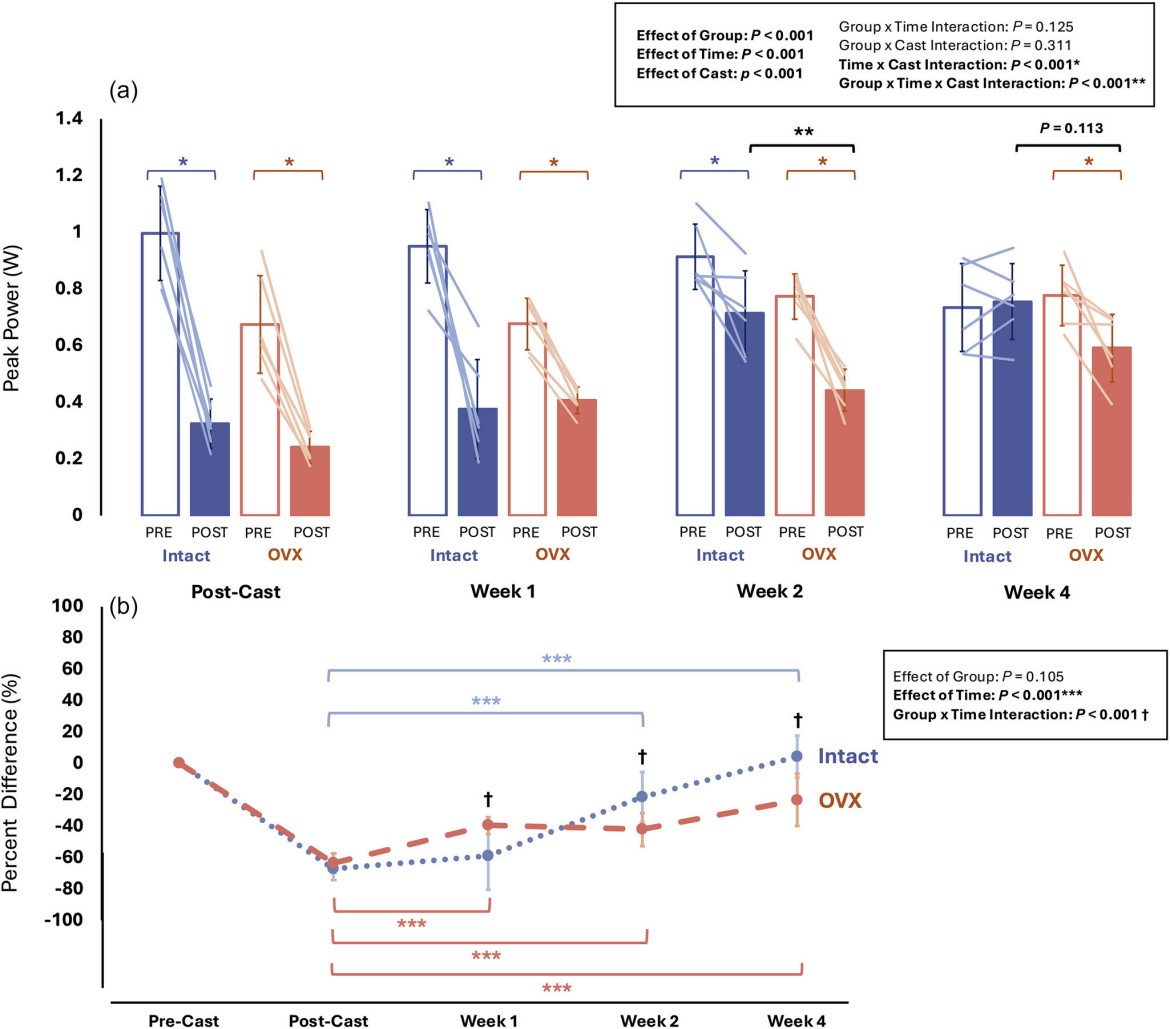
Differences in plantar flexor peak power in intact (*n* = 24) and ovariectomized (OVX; *n* = 24) adult female rats across recovery time points following unilateral hindlimb immobilization in a shortened position. Data are displayed as means ± standard deviation. (a) Absolute pre‐ and post‐cast power values for intact and OVX rats at post‐cast, 1 week, 2 weeks and 4 weeks of recovery (*n* = 6/group per time point). Horizontal lines represent the decrease in pre‐ to post‐cast power values for individual rats. Statistical comparisons were performed using a three‐way ANOVA (Group × Time × Cast). *Significant pre–post difference within a group at that time point (*P < *0.05). **Significant difference between intact and OVX post‐cast values at that time point (*P < *0.05). (b) Percentage difference in peak power from pre‐cast to post‐cast for each rat across the same recovery time points. Statistical comparisons were performed using a two‐way ANOVA (Group × Time) on percentage difference values. †Significant difference between intact and OVX groups percentage difference at that time point (*P < *0.05). ***Significant difference from the post‐cast time point when pre‐ and post‐cast values are combined; purple bars represent intact group and orange bars represent OVX group (*P < *0.05).

Absolute peak plantar flexor power (Figure 7a) showed significant effects of Group (*P* < 0.001; η^2^ = 0.303), Time (*P* < 0.001; η^2^ = 0.269), and Cast (*P* < 0.001; η^2^ = 0.686), as well as significant Time × Cast (*P* < 0.001; η^2^ = 0.380) and Group × Time × Cast interactions (*P* < 0.001; η^2^ = 0.200). Overall, the OVX group exhibited lower pre‐cast peak power compared to the intact group (*P* < 0.001), indicating baseline group differences independent of immobilization. Percentage difference between pre‐ and post‐cast peak power (Figure 7b) showed a significant effect of Time (*P* < 0.001; η^2^ = 0.746) and a significant Group × Time interaction (*P* < 0.001; η^2^ = 0.378), indicating that the recovery of cast‐induced power loss differed between intact and OVX groups. Immediately following cast removal, intact and OVX groups experienced similarly large deficits in power compared to pre‐cast values (−67% and −63% from pre‐cast, respectively, *P <* 0.001). However, the recovery trajectory differed between groups such that, in the intact group, power increased steadily across the recovery period and by week 4 no longer differed from pre‐cast values (+4% from pre‐cast, *P *= 0.762). In contrast, the OVX group remained significantly impaired by week 4 (–23% from pre‐cast, *P =* 0.0102). Overall power in the OVX group was lower than the intact group at week 2 and week 4.

### 3.2 Architectural adaptations

#### 3.2.1 Soleus serial sarcomere number

**FIGURE 8:**
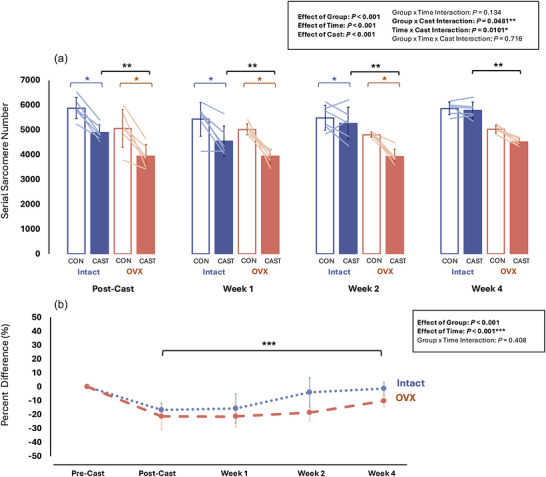
Differences in soleus serial sarcomere number (SSN) in intact (*n* = 24) and ovariectomized (OVX; *n* = 24) adult female rats following unilateral hindlimb immobilization in a shortened position. Data are displayed as means ± standard deviation. (a) Absolute soleus SSN values for the control (CON) and casted (CAST) legs of intact and OVX rats at post‐cast, 1 week, 2 weeks and 4 weeks of recovery (*n* = 6/group per time point). Horizontal lines represent the difference in CON and CAST SSN values for individual rats. Statistical comparisons for were performed using a three‐way ANOVA (Group × Time × Cast). *Significant CON–CAST difference at specific time points with groups combined (*P < *0.05). **Significant difference between intact and OVX CAST values averaged across time (*P < *0.05). (b) Percentage difference in soleus SSN between CON and CAST legs for each rat across the same recovery time points. Statistical comparisons were performed using a two‐way ANOVA (Group × Time) on percentage difference values. ***Significant difference from the post‐cast time point when CON and CAST values are combined; black bar represents groups combined (*P < *0.05).

Absolute soleus SSN (Figure 8a) was derived from measured soleus FL (Supporting information, Figure S7a) and soleus SL (Supporting information, Figure S8a) and showed significant main effects of Group (*P* < 0.001; η^2^ = 0.001), Time (*P* < 0.001; η^2^ = 0.214), and Cast (*P <* 0.001; η^2^ = 0.451), as well as significant Group × Cast (*P =* 0.0481; η^2^ = 0.048) and Time × Cast (*P =* 0.0101; η^2^ = 0.130) interactions, but no significant Group × Time × Cast interaction (*P* = 0.716; η^2^ = 0.017). Across all recovery time points, the OVX group exhibited substantially lower CON soleus SSN compared to the intact group (*P* < 0.001), indicating large baseline group differences independent of immobilization. Percentage difference between CON and CAST soleus SSN (Figure 8b) showed significant effects of Time (*P* < 0.001; η^2^ = 0.374) and Group (*P <* 0.001; η^2^ = 0.263), but no significant Group × Time interaction (*P* = 0.408; η^2^ = 0.069), indicating greater overall SSN deficits in the OVX groups but similar recovery trajectories between intact and OVX groups. Immediately following cast removal, intact and OVX groups exhibited comparable deficits in soleus SSN (–16% and –15% from CON, respectively; both *P <* 0.001). Soleus SSN increased across recovery in both groups. However, the OVX group maintained lower SSN values overall compared to the intact group (*P <* 0.001) and retained a statistically significant deficit at week 4 (–10% from CON; *P <* 0.001) whereas intact rats fully restored SSN by week 4 (–1% from CON; *P* = 0.146). Thus, both groups followed a similar recovery trajectory, but the OVX group never fully reached the SSN levels of the intact group.

#### 3.2.2 Soleus wet weight

**FIGURE 9:**
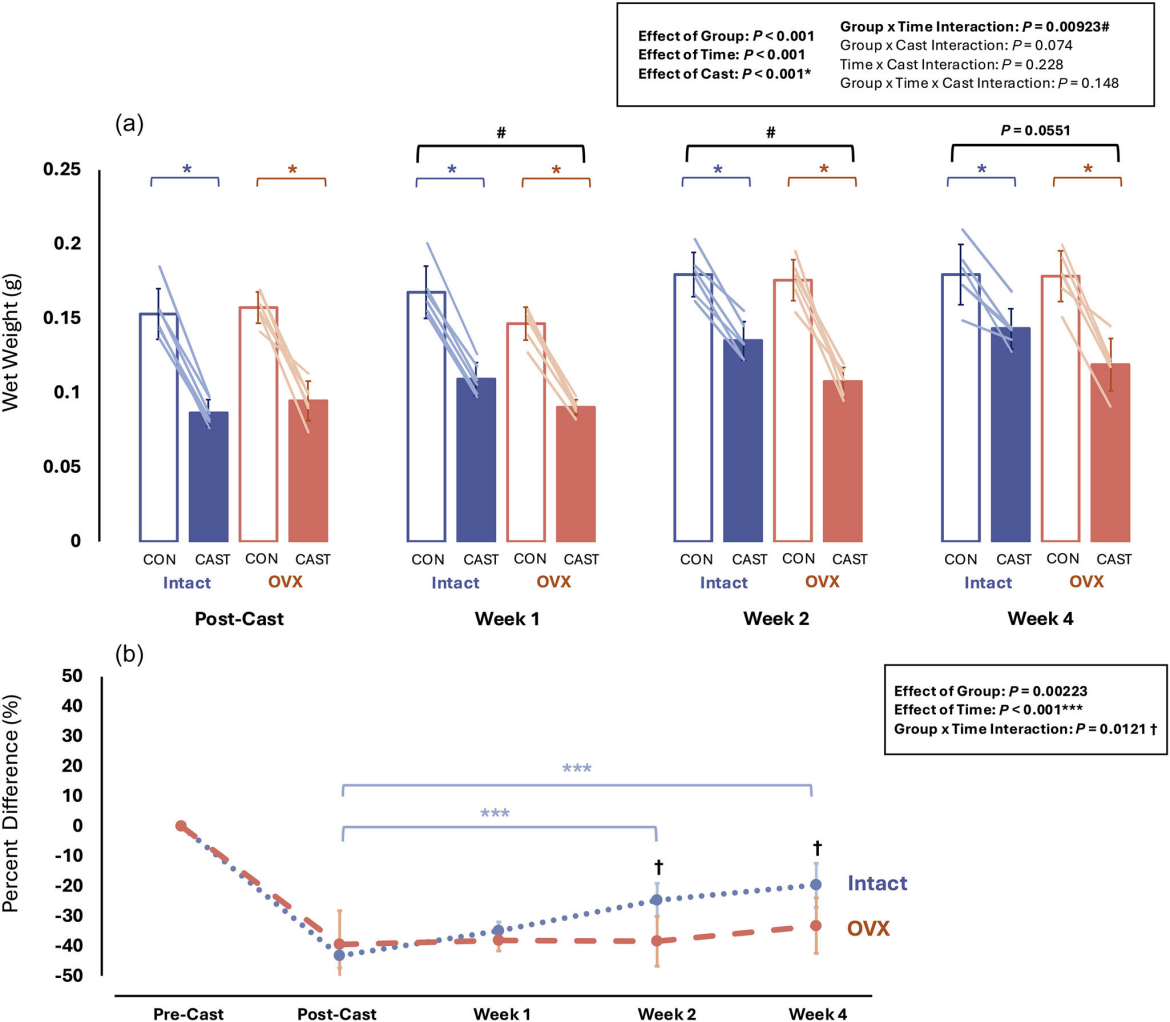
Differences in soleus wet weight (WW) in intact (*n* = 24) and ovariectomized (OVX; *n* = 24) adult female rats following unilateral hindlimb immobilization in a shortened position. Data are displayed as means ± standard deviation. (a) Absolute soleus WW values for the control (CON) and casted (CAST) legs of intact and OVX rats at post‐cast, 1 week, 2 weeks and 4 weeks of recovery (*n* = 6/group per time point). Horizontal lines represent the difference in CON and CAST WW values for individual rats. Statistical comparisons for were performed using a three‐way ANOVA (Group × Time × Cast). *Significant CON–CAST difference averaged across time with groups combined (*P < *0.05). #Significant difference between intact and OVX groups at that time point with CON and CAST values combined (*P < *0.05). (b) Percentage difference in soleus WW between CON and CAST legs for each rat across the same recovery time points. Statistical comparisons were performed using a two‐way ANOVA (Group × Time) on percentage difference values. †Significant difference between intact and OVX groups percentage difference at that time point (*P < *0.05). ***Significant difference from the post‐cast time point when CON and CAST values are combined; purple bars represent intact group (*P < *0.05).

Absolute soleus wet weight (Figure 9a) showed significant effects of Group (*P* < 0.001; η^2^ = 0.146), Time (*P* < 0.001; η^2^ = 0.534), and Cast (*P* < 0.001; η^2^ = 0.832), with a significant Group × Time (*P* = 0.00923; η^2^ = 0.133) interaction, but no significant Group × Time × Cast interaction (*P* = 0.148; η^2^ = 0.064). Percentage difference between CON and CAST soleus wet weight (Figure 9b) showed significant effects of Group (*P* = 0.00223; η^2^ = 0.209) and Time (*P* < 0.001; η^2^ = 0.422), and a significant Group × Time interaction (*P* = 0.0121; η^2^ = 0.243), indicating that the recovery of cast‐induced soleus wet weight loss differed between intact and OVX groups. Immediately following cast removal, intact and OVX groups exhibited comparable deficits in soleus wet weight (–43% and –39% from CON, respectively *P <* 0.001). Soleus wet weight increased across recovery in the intact group, such that by weeks 2 and 4, the percentage change differed significantly from the post‐cast time point (−24% and −19% from CON, respectively *P* < 0.001). However, the OVX group did not recover across time points, such that the percentage change at weeks 1, 2 and 4 did not differ significantly from the post‐cast time point (−38%, −38%, −33% from CON, respectively *P* > 0.05).

Direct comparisons revealed significant differences between intact and OVX groups at weeks 2 and 4 (both *P* = 0.00221), with both groups showing similar reductions in soleus wet weight immediately after cast removal, followed by partial recovery in intact rats and minimal recovery in OVX rats; however, neither group returned to control‐leg values by week 4.

#### 3.2.3 Soleus physiological cross‐sectional area

**FIGURE 10:**
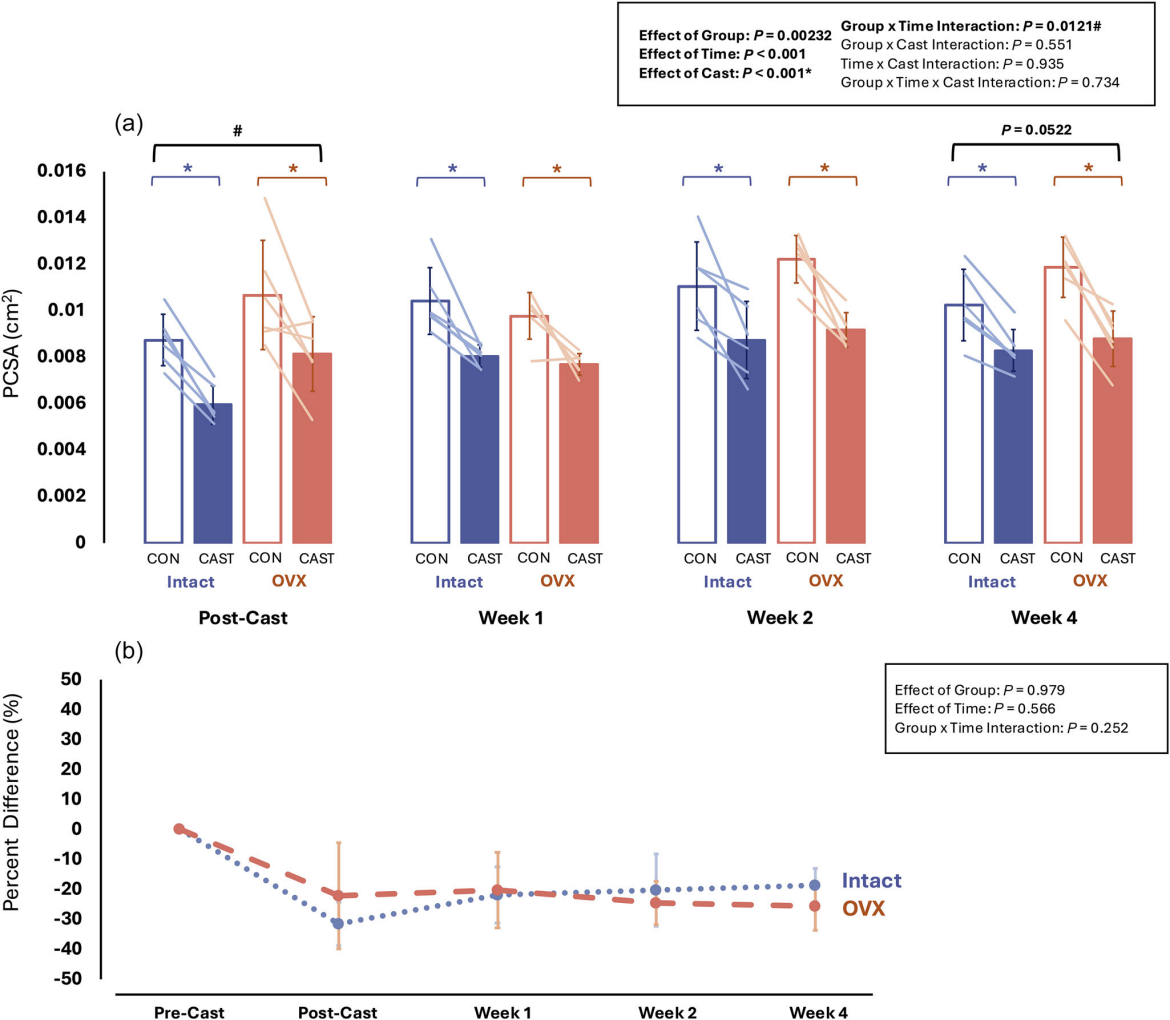
Differences in soleus physiological cross‐sectional area (PCSA) in intact (*n* = 24) and ovariectomized (OVX; *n* = 24) adult female rats following unilateral hindlimb immobilization in a shortened position. Data are displayed as means ± standard deviation. (a) Absolute soleus PCSA values for the control (CON) and casted (CAST) legs of intact and OVX rats at post‐cast, 1 week, 2 weeks and 4 weeks of recovery (*n* = 6/group per time point). Horizontal lines represent the difference in CON and CAST PCSA values for individual rats. Statistical comparisons were performed using a three‐way ANOVA (Group × Time × Cast). *Significant CON–CAST difference averaged across time with groups combined (*P < *0.05). #Significant difference between intact and OVX groups at that time point with CON and CAST values combined (*P < *0.05). (b) Percentage difference in soleus PCSA between CON and CAST legs for each rat across the same recovery time points. Statistical comparisons were performed using a two‐way ANOVA (Group × Time) on percentage difference values.

Absolute soleus PCSA (Figure 10a) showed a significant effect of Group (*P* = 0.00232; η^2^ = 0.113), Time (*P* < 0.001; η^2^ = 0.273), and Cast (*P* < 0.001; η^2^ = 0.523), with a significant Group × Time interaction (*P* = 0.0121; η^2^ = 0.127), but no significant Group × Time × Cast interaction (*P* = 0.734; η^2^ = 0.016). Intact and OVX soleus PCSA differed overall at the post‐cast time point (*P *< 0.001) and remain significantly different at the week 4 time point (*P* = 0.0522). Percentage difference between CON and CAST soleus PCSA (Figure 10b) showed no significant effects of Group (*P* = 0.979; η^2^ = 0.000), Time (*P* = 0.560; η^2^ = 0.050), or a Group × Time interaction (*P* = 0.252; η^2^ = 0.096), indicating that changes in soleus PCSA immediately following cast removal were similar between intact and OVX groups (−31% and −22% from CON, respectively *P* < 0.05) and remained relatively stable across recovery.

#### 3.2.4 Medial gastrocnemius serial sarcomere number

**FIGURE 11:**
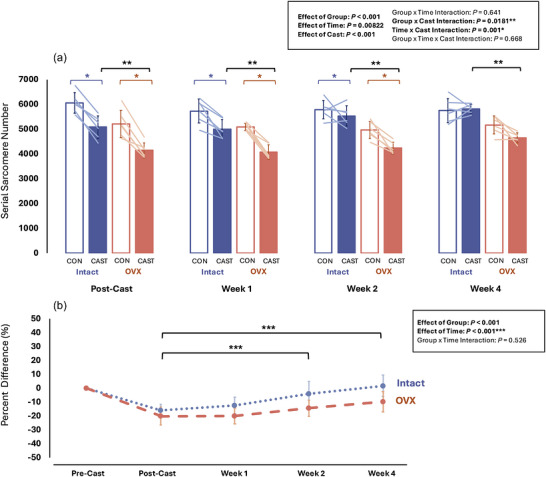
Differences in medial gastrocnemius (MG) serial sarcomere number (SSN) in intact (*n* = 24) and ovariectomized (OVX; *n* = 24) adult female rats following unilateral hindlimb immobilization in a shortened position. Data are displayed as means ± standard deviation. (a) Absolute MG SSN values for the control (CON) and casted (CAST) legs of intact and OVX rats at post‐cast, 1 week, 2 weeks and 4 weeks of recovery (*n* = 6/group per time point). Horizontal lines represent the difference in CON and CAST SSN values for individual rats. Statistical comparisons were performed using a three‐way ANOVA (Group × Time × Cast). *Significant CON–CAST difference at specific time points with groups combined (*P < *0.05). **Significant difference between intact and OVX CAST values averaged across time (*P < *0.05). (b) Percentage difference in MG SSN between CON and CAST legs for each rat across the same recovery time points. Statistical comparisons were performed using a two‐way ANOVA (Group × Time) on percentage difference values. ***Significant difference from the post‐cast time point when CON and CAST values are combined; black bars represent groups combined (*P < *0.05).

Absolute MG SSN (Figure 11a) was derived from measured MG FL (Supporting information, Figure S9a) and MG SL (Supporting information, Figure S10a), and showed significant main effects of Group (*P <* 0.001; η^2^ = 0.642), Time (*P =* 0.00822; η^2^ = 0.136), and Cast (*P <* 0.001; η^2^ = 0.483), as well as significant Group × Cast (*P =* 0.0181; η^2^ = 0.068) and Time × Cast interactions (*P <* 0.001; η^2^ = 0.176), but no Group × Time × Cast interaction (*P* = 0.668; η^2^ = 0.019). Across all time points, the OVX group exhibited substantially lower CON MG SSN compared to the intact group (*P* < 0.001), indicating large baseline group differences independent of immobilization. Percentage difference between CON and CAST MG SSN (Figure 11b) showed significant effects of Time (*P <* 0.001; η^2^ = 0.459) and Group (*P <* 0.001; η^2^ = 0.320), but no significant Group × Time interaction (*P <* 0.001; η^2^ = 0.054), indicating greater overall SSN deficits in the OVX group but similar recovery trajectories between intact and OVX groups. Immediately following cast removal, intact and OVX groups exhibited comparable deficits in MG SSN (–16% and –20% from CON, respectively *P <* 0.001). Medial gastrocnemius SSN increased across recovery in both groups. However, the OVX group maintained lower SSN values overall compared to the intact group (*P <* 0.001), and retained a small, but not statistically significant deficit at week 4 (–10% from CON, *P* = 0.152), whereas the intact group fully restored SSN by week 4 (+1% from CON, *P* = 0.863). Thus, both groups followed a similar recovery trajectory, but the OVX group never fully reached the SSN levels of the intact group.

#### 3.2.5 Medial gastrocnemius wet weight

**FIGURE 12:**
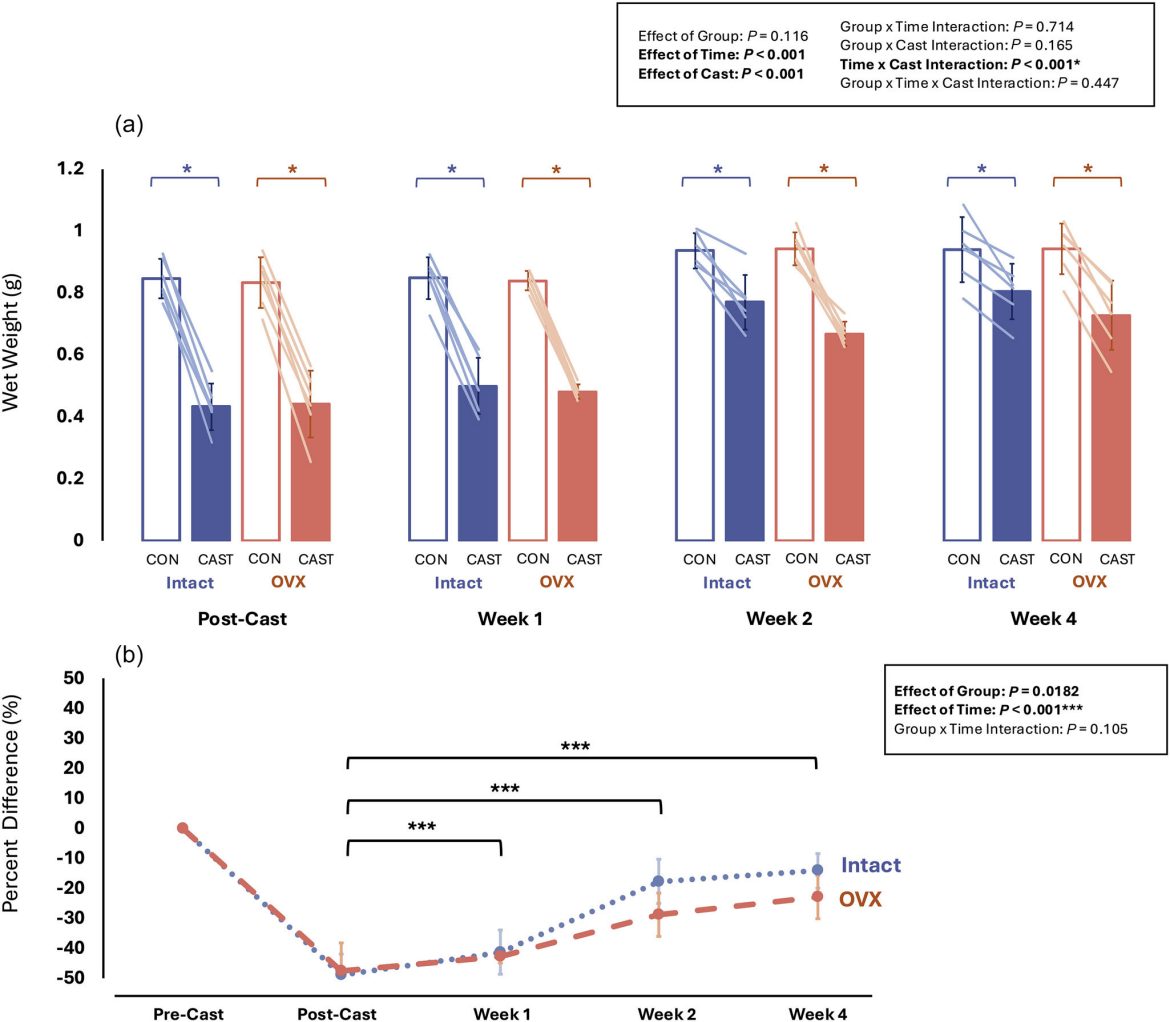
Differences in medial gastrocnemius (MG) wet weight (WW) in intact (*n* = 24) and ovariectomized (OVX; *n* = 24) adult female rats following unilateral hindlimb immobilization in a shortened position. Data are displayed as means ± standard deviation. (a) Absolute MG WW values for the control (CON) and casted (CAST) legs of intact and OVX rats at post‐cast, 1 week, 2 weeks and 4 weeks of recovery (*n* = 6/group per time point). Horizontal lines represent the difference in CON and CAST WW values for individual rats. Statistical comparisons were performed using a three‐way ANOVA (Group × Time × Cast). *Significant CON–CAST difference at specific time points with groups combined (*P < *0.05). (b) Percentage difference in MG WW between CON and CAST legs for each rat across the same recovery time points. Statistical comparisons were performed using a two‐way ANOVA (Group × Time) on percentage difference values. ***Significant difference from the post‐cast time point when CON and CAST values are combined, with black bars representing groups combined (*P < *0.05).

Absolute MG wet weight (Figure 12a) showed significant effects of Time (*P* < 0.001; η^2^ = 0.645) and Cast (*P* < 0.001; η^2^ = 0.806), a significant Time × Cast (*P* < 0.001; η^2^ = 0.305) interaction, but no significant Group × Time × Cast interaction (*P* = 0.447; η^2^ = 0.033). Percentage difference between CON and CAST MG wet weight (Figure 12b) showed significant effects of Group (*P* = 0.0182; η^2^ = 0.132) and Time (*P* < 0.001; η^2^ = 0.793), but no significant Group × Time interaction (*P* = 0.105; η^2^ = 0.141), indicating greater overall MG wet weight deficits in the OVX group but similar recovery trajectories between intact and OVX groups. Immediately following cast removal, intact and OVX groups exhibited comparable deficits in MG wet weight (–49% and –47% from CON, respectively *P <* 0.001). Across recovery, MG wet weight progressively increased toward CON values, with significant differences from the post‐cast time point observed at the week 1, 2 and 4 recovery time points when groups were combined (*P* < 0.05, respectively).

#### 3.2.6 Medial gastrocnemius physiological cross‐sectional area

**FIGURE 13:**
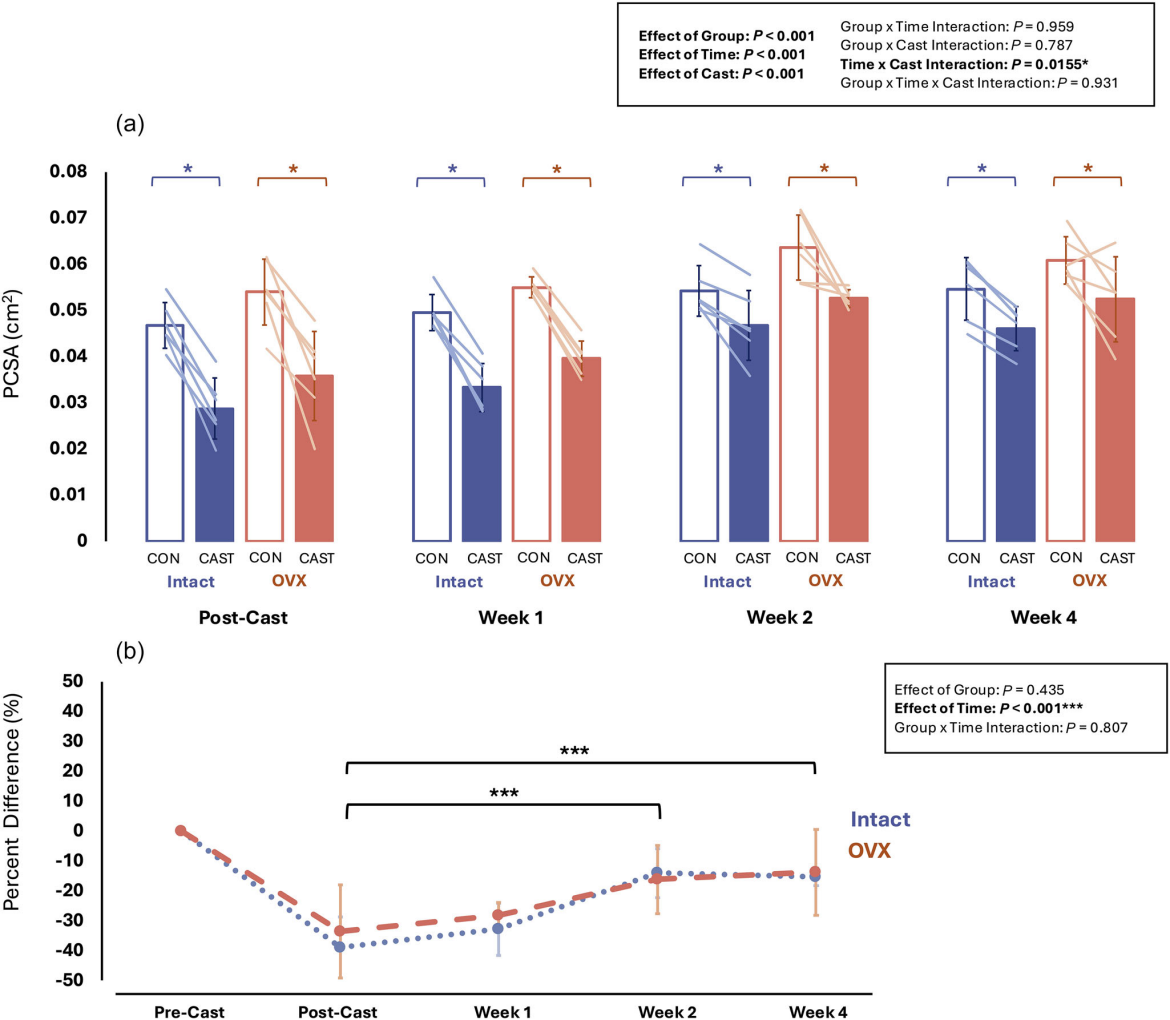
Differences in medial gastrocnemius (MG) physiological cross‐sectional area (PCSA) in intact (*n* = 24) and ovariectomized (OVX; *n* = 24) adult female rats following unilateral hindlimb immobilization in a shortened position. Data are displayed as means ± standard deviation. (a) Absolute MG PCSA values for the control (CON) and casted (CAST) legs of intact and OVX rats at post‐cast, 1 week, 2 weeks and 4 weeks of recovery (*n* = 6/group per time point). Horizontal lines represent the difference in CON and CAST PCSA values for individual rats. Statistical comparisons for were performed using a three‐way ANOVA (Group × Time × Cast). *Significant CON–CAST difference at specific time points with groups combined (*P < *0.05). (b) Percentage difference in MG PCSA between CON and CAST legs for each rat across the same recovery time points. Statistical comparisons were performed using a two‐way ANOVA (Group × Time) on percentage difference values. ***Significant difference from the post‐cast time point when CON and CAST values are combined; black bars represent groups combined (*P < *0.05).

Absolute MG PCSA (Figure 13a) showed a significant effect of Group (*P* < 0.001; η^2^ = 0.268), Time (*P* < 0.001; η^2^ = 0.508), and Cast (*P* < 0.001; η^2^ = 0.574), with a significant Time × Cast interaction (*P* = 0.0155; η^2^ = 0.122), but no significant Group × Time × Cast interaction (*P* = 0.931; η^2^ = 0.006). The intact group had lower MG PCSA values overall across all time points compared to the OVX group (*P *< 0.001). Percentage difference in PCSA between the CON and CAST MG (Figure 13b) showed a significant effect of Time (*P* < 0.001; η^2^ = 0.503), but no significant effect of Group (*P* = 0.435; η^2^ = 0.015), or a Group × Time interaction (*P* = 0.807; η^2^ = 0.024), indicating that deficits in MG PCSA between CON and CAST legs recovered similarly across time. Immediately following cast removal, intact and OVX groups exhibited comparable deficits in MG PCSA (–39% and –33% from CON, respectively *P <* 0.001). Across recovery, PCSA of the MG progressively increased toward CON values, with significant differences from the post‐cast time point observed at the week 2 and 4 recovery time points when groups were combined (*P* < 0.001).

## 4 DISCUSSION

The purpose of this study was to examine the effects of oestrogen on architectural and mechanical adaptations of the plantar flexors following immobilization in a shortened position and during subsequent recovery. Using a unilateral hindlimb casting model of intact and OVX adult female rats, we found that 2 weeks of immobilization in a shortened muscle position resulted in 13–50% decreases in FL, SSN, PCSA and muscle wet weight alongside 20–65% reductions in active peak torque, normalized peak torque, power and velocity, in both groups. During recovery, OVX rats exhibited blunted restoration of both architectural and mechanical measures compared to the intact rats, indicating that oestrogen deficiency did not modify the acute response to immobilization but indeed influenced the time course of recovery of architectural and mechanical features following disuse.

### 4.1 Baseline differences in muscle morphology and function between groups

#### 4.1.1 Baseline differences in muscle morphology

Consistent with previous findings (Vidal et al., 2020), OVX rats exhibited ∼13% lower SSN in both the soleus and MG of the control limb (Figures 8a, 11a), likely driving the shorter FL observed (∼12%; Supporting information, Figures S7a, S9a) as compared with the intact group. Despite shorter fascicles, OVX rats had 10–13% greater PCSA (Figures 10a, 13a) compared to intact rats, consistent with previous work indicating increased muscle size following ovariectomy (Greising et al., 2011; Lara‐García et al., 2011; Moran et al., 2006). Oestrogen deficiency has been shown to promote lipid accumulation and adipogenic gene expression (Jackson et al., 2013; Stubbins et al., 2012), suggesting that the increased muscle size in OVX rats may be a result of greater intramyocellular lipid content rather than increased myofibrillar protein (Collins et al., 2019; Greising et al., 2011; Lara‐García et al., 2011; Moran et al., 2006). To isolate intrinsic contractile quality and remove the influence of muscle size, torque was normalized to PCSA. When normalized to PCSA, torque (peak torque/PCSA) was 10–17% lower in OVX rats compared to intact rats, despite similar absolute torque at baseline (Figures 4a, 6a), likely because OVX rats had greater PCSA at baseline (as discussed above).

#### 4.1.2 Baseline differences in power and velocity

Peak power did not differ between groups at baseline despite OVX rats exhibiting reduced maximal velocity (−46%; Supporting information, Figure S4a), and velocity at peak power (−33%; Supporting information, Figure S5a). This reflects a leftward shift of the force–velocity relationship, whereby reductions in velocity shifted peak power toward higher relative force (Hill, 1938; Seow, 2013; Hinks et al., 2022) in OVX compared to intact rats. The reduced velocity in OVX rats is consistent with oestrogen's role in decreases myosin regulatory light chain (RLC) phosphorylation and impairing calcium–troponin interactions that govern cross‐bridge kinetics (Lai et al., 2016; Moran et al., 2006, 2007; McGoldrick et al., 1998; Wattanapermpool & Reiser, 1999), though model‐dependent variability has been reported (Greising et al., 2011; Hinks & Power, 2024). The phosphorylation of RLC increases the proximity of myosin cross‐bridges to actin and reduces interfilament spacing, accelerating the rate of force development and increases calcium sensitivity (Colson et al., 2010); thus reduced RLC phosphorylation would slow cross‐bridge cycling, which is the primary determinant of velocity (Edman, 2014). The lower SSN of OVX rats at baseline would further constrain velocity because fewer sarcomeres arranged in series requires each sarcomere to undergo a greater excursion for a given change in muscle length, thereby limiting the maximum shortening speed (Hinks et al., 2022). Therefore, reductions in velocity at baseline in OVX rats were likely driven by oestrogen deficiency‐mediated changes in cross‐bridge kinetics and decreased baseline SSN. Despite these velocity deficits, power remained equivalent to the intact group, likely because OVX rats compensated through enhanced force generation, potentially facilitated by a stiffer muscle–tendon unit.

### 4.2 Similar magnitude of disuse‐induced loss in mechanical function and muscle morphology following casting in both OVX and intact rats

#### 4.2.1 Disuse‐induced loss of muscle morphological measures

Following 2 weeks of immobilization, both groups exhibited similar deficits in muscle wet weight (−40%; Figures 9b, 12b), PCSA (−25%; Figures 10b, 13b), FL (−13%; Supporting information, Figures S7b, S9b), active peak torque (−50%; Figures 3b, 5b), normalized peak torque (−20%; Figures 4b, 6b), and peak power (−65%; Figure 7b), aligning with previous work showing comparable reductions between intact and OVX females during 10–14 days of disuse via hindlimb suspension (Fisher et al., 1998; Brown et al., 2005; McClung et al., 2006; Sitnick et al., 2006; Rosa‐Caldwell et al., 2023). We extended these findings by assessing SSN remodelling for the first time in an ovariectomized immobilization model. The ∼18% SSN loss (Figures 8b, 11b) was similar between groups and muscles, and this loss is consistent with established literature following immobilization in a shortened muscle position (Williams & Goldspink, 1978; Hinks & Power, 2024, 2025). With this and similar models, it has been suggested that extreme unloading drives all animals toward a similar physiological limit of muscle loss, potentially masking hormone‐dependent effects at the post‐cast time point (Rosa‐Caldwell et al., 2023). This adaptation (immediate loss of SSN) likely aims to restore resting sarcomere length of the muscle toward an optimal range for force production at the habituated immobilized joint angle (Hinks et al., 2022).

#### 4.2.2 Disuse‐induced increases in passive tension

Immobilization increased passive torque (∼240–300%; Supporting information, Figures S2b, S3b) in both groups, reflecting substantial increases in muscle stiffness (Hinks & Power, 2024, 2025; Tabary et al., 1972; Williams & Goldspink, 1978). However, OVX rats exhibited 78–98% greater passive torque post‐cast than intact rats. Since SSN loss was similar between groups, architectural changes alone cannot explain this difference. Instead, oestrogen deficiency may have promoted greater extracellular matrix accumulation, fibrosis and collagen cross‐linking (Greising et al., 2011; Hansen & Kjaer, 2016; Kjaer, 2004; Moran et al., 2006), resulting in hormone‐specific effects on passive stiffness independent of longitudinal remodelling.

### 4.3 Oestrogen deficiency influenced recovery of muscle morphology

#### 4.3.1 Recovery of radial morphological measures

Muscle wet weight and PCSA recovery were blunted in OVX rats. By week 4, intact rats recovered wet weight to 80–86% of baseline in the soleus and MG, whereas OVX rats reached only 66–77% (Figures 9b, 12b). Recovery of PCSA was muscle‐dependent, with greater deficits in the OVX soleus (–26% vs. –19% intact) but similar recovery between groups in the MG (–14% in both groups; Figures 10b, 13b). Better MG recovery in both groups likely reflects its biarticular nature, allowing earlier re‐engagement during re‐ambulation compared to the monoarticular soleus (Cleland, 1867; Woittiez et al., 1985). These findings align with prior work consistently reporting delayed or incomplete recovery of muscle mass in OVX animals (Brown et al., 2005; McClung et al., 2006; Sitnick et al., 2006). Therefore, the magnitude of atrophy following severe disuse is similar between groups; however, oestrogen deficiency disproportionately impairs the recovery of muscle mass during reloading.

#### 4.3.2 Recovery of longitudinal morphological measures

Longitudinal morphological adaptations recovered more rapidly than radial measures in both groups. Intact rats recovered fully and surpassed control values for FL by week 2, whereas OVX rats remained impaired with consistently greater deficits (Supporting information, Figures S7b, S9b). By week 4, intact rats achieved full SSN recovery in both muscles, whereas OVX rats retained ∼10% deficits (Figures 8b, 11b). This faster recovery of longitudinal over radial increases in muscle size is consistent with the concept that serial sarcomerogenesis is strongly stimulated by stretch–shortening cycles and increased range of motion (Williams & Goldspink, 1978; Hinks & Power, 2024), whereas muscle mass and PCSA recovery typically require greater mechanical loading, such as resistive exercise (Hinks et al., 2022; Kruse et al., 2021). This also may indicate that the neuromuscular system may prioritize restoration of muscle length and excursion capacity before rebuilding contractile tissue in parallel, potentially due to the functional importance of longitudinal architecture for power production and movement efficiency (Lieber et al., 2017; Kruse et al., 2021; Hinks et al., 2022). Delayed recovery of radial and longitudinal measures in OVX rats may, in part, be due to altered protein degradation pathways in the absence of oestrogen. Oestrogen modulates ‘atrophy genes’ encoding ubiquitin ligases involved in muscle protein breakdown (Bodine et al., 2001; Gomes et al., 2001), and OVX mice have been shown to exhibit elevated MuRF1 and MAFbx expression (Rogers et al., 2010). Oestrogen may also influence recovery through protective effects against cellular stress and apoptosis via heat shock proteins (Paroo et al., 2002; Lee et al., 2007; Bombardier et al., 2013), contributing to prolonged catabolic signalling and the impaired restoration of muscle mass observed in OVX rats.

### 4.4 Oestrogen deficiency led to an incomplete recovery of mechanical function

#### 4.4.1 Recovery of active peak torque

Intact rats demonstrated substantially greater restoration of active peak torque during recovery, with significant improvements from post‐cast at both time points, whereas OVX rats did not (Figure 3a,b). Importantly, the persistent torque deficits observed in OVX rats occurred despite relatively similar recovery of PCSA between groups (see Figures 10b, 13b), but more closely aligning with recovery of soleus wet weight (Figure 9b). This dissociation between recovery of PCSA and torque suggests torque production is not fully explained by muscle size alone, consistent with studies showing oestrogen deficiency impairs force production independent of muscle mass (Moran et al., 2006; Greising et al., 2011). Reduced myosin RLC phosphorylation in oestrogen‐deficient muscle may reduce cross‐bridge attachment rates (Colson et al., 2010; Gregorich et al., 2016; Kararigas et al., 2012), impairing force generation without altering muscle size. Notably, the intact rats exceeded pre‐cast torque values by week 4. Rather than reflecting true super‐compensatory mechanisms, this observation likely reflects continued maturation during the study window as rats in this age range may continue to gain body mass and force‐producing capacity over time (Brower et al., 2015; Hinks & Power, 2024; Sengupta, 2013; Turturro et al., 1999). Supporting this interpretation, body mass progressively increased throughout recovery, with intact rats exceeding baseline body mass by ∼10% at week 4 (Supporting information, Figure S11). Therefore, while the present model remains physiologically relevant for studying recovery following immobilization, future studies may benefit from the use of other models such as the 4‐vinylcyclcohexene diepoxide (VCD) model (Hubbard et al., 2023; Mashouri et al., 2025) or senescent rodents to more closely relate adaptative changes to the hormonal milieu of old age.

#### 4.4.2 Recovery of peak power and velocity

Recovery of peak power differed substantially between groups whereby at week 4, intact rats had recovered to baseline (+3%), whereas OVX rats remained impaired (−24%; Figure 7b). The apparent dissociation between recovery of velocity‐related variables and lack of recovery of peak power in OVX rats likely reflects how torque–velocity behaviour is shaped by force‐generating capacity of the muscle, SSN and stiffness of the muscle–tendon unit. Because power is the product of torque and velocity (Hill, 1938; Seow, 2013), the recovery of velocity measures and SSN in OVX rats indicates that longitudinal remodelling was re‐established, facilitating faster shortening at peak power. However, the continued impairment in peak power likely reflects incomplete recovery of torque, potentially driven by deficits in contractile function and limited radial growth. This pattern suggests a preferential restoration of longitudinal over radial adaptations in muscle. In contrast, intact rats demonstrated recovery of torque alongside SSN, consistent with greater radial growth and resulting in full restoration of peak power by week 4.

Conversely, architectural factors may also contribute to the blunted recovery of power in the OVX group. Reductions in SSN would be expected to constrain whole‐muscle velocity by reducing the number of sarcomeres operating in series, and data in humans indicate that shorter fascicles explain a substantial portion of reduced velocity (Reeves & Narici, 2003). However, in the present study, SSN recovery improved ∼80% in both groups by week 4 (see Figure 11b), suggesting that incomplete SSN recovery alone is unlikely to fully explain persistent power impairments; instead, increased passive tension may play a role. OVX rats demonstrated higher passive torque across most time points, which would not contribute to enhancing velocity, which in turn would reduce power output at the joint level even when architectural measures appear recovered (Earp et al., 2014; Hinks et al., 2022).

### 4.5 Methodological considerations and future directions

Minimal recovery was observed across many measures at week 1 following cast removal in both groups, potentially reflecting continued disuse as rats would hold the muscle at a shortened length for several days post‐cast removal. Since both groups showed similarly blunted recovery at this time point, this effect is unlikely to account for the divergent trajectories at later time points. Nevertheless, the inability to quantify limb use during early recovery may have reduced sensitivity to detect subtle group differences. Additionally, spontaneous activity levels were not measured. Since voluntary reloading is a key stimulus for muscle remodelling and OVX rodents show lower spontaneous cage activity (Greising et al., 2011), unmeasured activity differences could have contributed to the blunted functional recovery observed in OVX rats. Future studies incorporating activity monitoring or controlled reambulation protocols would strengthen interpretation.

We did not perform in vivo architectural measurements on the uncasted limb prior to or during immobilization. However, prior work using the same immobilization model reported no changes in soleus fascicle length in the uncasted limb during casting (Hinks et al., 2023), suggesting any compensatory remodelling is likely modest. Additionally, pre‐cast normalized torque values were estimated using terminal contralateral PCSA measurements, as direct assessment of PCSA at the time of baseline mechanical testing was not possible without terminal tissue collection. Therefore, normalized torque should be interpreted alongside absolute mechanical and architectural measures. Nevertheless, inclusion of normalized torque provided additional context regarding force production relative to muscle size, particularly given baseline morphological differences between intact and OVX rats.

Increases in SL (+2–8%) observed following casting are owing to the methods used to determine SSN (Supporting information, Figures S8b, S10b). We fixed the ankle angle at 90° rather than at an optimal muscle length (*L*_0_) for force production. As shifts in *L*_0_ depend on the magnitude of imposed mechanical stimuli (Chen et al., 2007) sarcomeres may have been relatively stretched at the fixed joint angle, influencing measured SL. While fixing at *L*_0_ would be ideal, SSN remains a valid indicator of longitudinal remodelling because it integrates independent measures of FL and SL and is less influenced by fixation‐related effects (Hinks & Power, 2024; Rilling et al., 2025). Additionally, ovariectomy reduces all gonadal hormones including oestrogen, progesterone and testosterone (Alagwu & Nneli, 2005; Moran et al., 2006). Circulating hormone levels were not measured, oestrous cycle stage was not controlled in intact rats, and intact animals did not undergo sham surgery. While transient oestrogen fluctuations at testing could have contributed variability (Ajayi & Akhigbe, 2020), these factors are unlikely to account for the magnitude of observed group differences.

### 4.6 Conclusion

Here, we show for the first time that ovarian hormone deficiency impairs the recovery of longitudinal muscle architecture, specifically SSN, following immobilization, despite similar initial losses between intact and OVX rats. However, during recovery, clear group differences emerged. Intact rats demonstrated restoration of active peak torque and peak power, whereas OVX rats exhibited persistently blunted recovery of mechanical function despite broadly similar recovery of PCSA. Instead, impaired recovery of torque was observed along with deficits in muscle wet weight, indicating impaired restoration of radial contractile tissue in oestrogen‐deficient muscle. Although SSN recovered more rapidly than radial measures in both groups, OVX rats retained residual SSN deficits that also coincided with sustained impairments in torque and power production. Together, these findings indicate that ovarian hormone deficiency disrupts the coordinated recovery of muscle architecture and contractile mechanics following disuse. This work establishes the previously unexplored adaptations of SSN, which suggests hormone‐sensitive determinant of functional recovery and highlights longitudinal remodelling as a critical contributor to the impaired mechanical recovery observed in oestrogen‐deficient states.

## AUTHOR CONTRIBUTIONS

Alexandra Q. Kirkup: Conceptualization, methodology, investigation, formal analysis, data curation, project administration, figure preparation, and writing—original draft. Amelia Rilling: Investigation, formal analysis, and writing—review and editing. Alexander M. Zero: Formal analysis and writing—review and editing. Geoffrey A. Power: Funding acquisition, methodology, resources, and writing—review and editing. All authors have read and approved the final version of this manuscript and agree to be accountable for all aspects of the work in ensuring that questions related to the accuracy or integrity of any part of the work are appropriately investigated and resolved. All persons designated as authors qualify for authorship, and all those who qualify for authorship are listed.

## CONFLICT OF INTEREST

None declared.

## GENERATIVE AI STATEMENT

Generative AI tools were not used in the preparation of this manuscript.

## Supporting information



Figure S1. Representative photograph of the custom 3D printed cast used to immobilize the left hindlimb of female rats.
Figure S2. Differences in plantar flexor passive tension at a 90° ankle angle.
Figure S3. Differences in plantar flexor passive tension at a 70° ankle angle.
Figure S4. Differences in maximum plantar flexor velocity.
Figure S5. Differences in optimal plantar flexor velocity.
Figure S6. Differences in optimal plantar flexor torque.
Figure S7. Differences in soleus fascicle length (FL).
Figure S8. Differences in soleus sarcomere length (SL).
Figure S9. Differences in medial gastrocnemius (MG) fascicle length (FL).
Figure S10. Differences in medial gastrocnemius (MG) sarcomere length (SL).
Figure S11. Differences in body mass.

## Data Availability

Supporting data are available upon request.
