# Supplementary material for: Alterations to longitudinal muscle morphology and mechanical function following immobilization and recovery in female ovariectomized and intact rats
Source: Exp Physiol. 2026 Jul 11:10.1113/EP093152. Online ahead of print. doi: 10.1113/EP093152 (PMC13394535; doi:10.1113/EP093152)
Supplement: Supplementary file 1 — Figure S1. Representative photograph of the custom 3D printed cast used to immobilize the left hindlimb of female rats. Figure S2. Differences in plantar flexor passive tension at a 90° ankle angle. Figure S3. Differences in plantar flexor passive tension at a 70° ankle angle. Figure S4. Differences in maximum plantar flexor velocity. Figure S5. Differences in optimal plantar flexor velocity. Figure S6. Differences in optimal plantar flexor torque. Figure S7. Differences in soleus fascicle length (FL). Figure S8. Differences in soleus sarcomere length (SL). Figure S9. Differences in medial gastrocnemius (MG) fascicle length (FL). Figure S10. Differences in medial gastrocnemius (MG) sarcomere length (SL). Figure S11. Differences in body mass. [file EPH-9999-0-s001.pdf]

Supplemental Material – Experimental Physiology Kirkup et al. (2026)

Alterations to longitudinal muscle morphology and mechanical function following immobilization and recovery in female ovariectomized and intact rats

Alexandra Q. Kirkup, Amelia Rilling, Alexander M. Zero & Geoffrey A. Power*

Department of Human Health Sciences, College of Biological Sciences, University of Guelph, 50 Stone Road East, Guelph, Ontario, Canada

***Correspondence**
Geoffrey A. Power PhD.
Neuromechanical **P**erformance **R**esearch **L**aboratory
Department of Human Health and Nutritional Sciences
College of Biological Sciences
University of Guelph, Ontario, Canada
Telephone: 1-519-824-4120 x53752
Email: gapower@uoguelph.ca

Supplemental Material

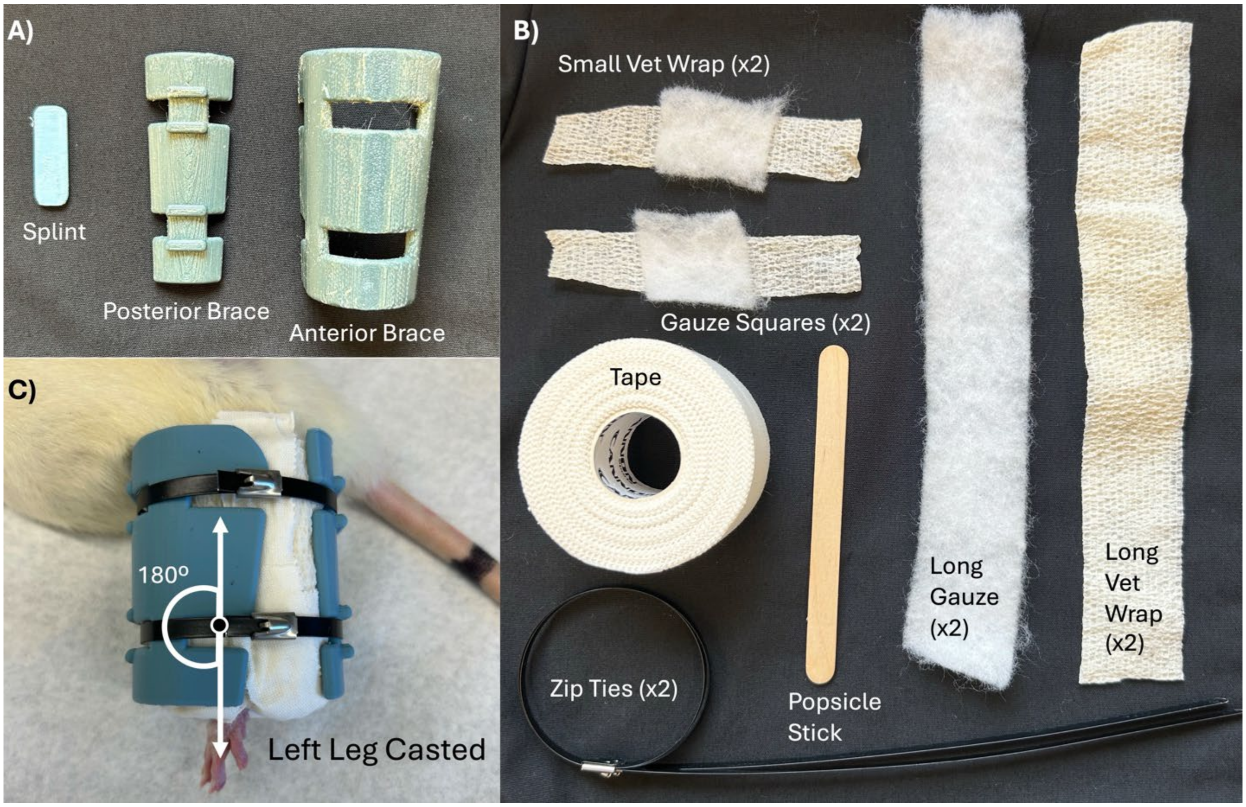

Supplemental Figure 1. **Representative photograph of the custom 3D printed cast used to immobilize the left hindlimb of female rats. (A)** Components of the 3D-printed cast system include a splint, posterior brace, and anterior brace. **(B)** Additional materials used to apply the cast include small and long vet wrap, gauze squares and strips, medical tape, zip ties, and a popsicle stick for structural support. **(C)** Fully applied cast spanning from the mid-femur to the foot, with the knee held in full extension and the ankle fixed in ~180° plantar flexion. This position places the soleus and medial gastrocnemius in a chronically shortened state. The right hindlimb remained free to allow normal cage activity.

2. Passive Tension at a 90 Degree Ankle Angle

Absolute passive tension at a 90° ankle angle (Supplemental Figure 2a) showed significant effects of Group ($p < 0.001$; $\eta^2 = 0.389$), Time ($p < 0.001$; $\eta^2 = 0.554$), and Cast ($p < 0.001$; $\eta^2 = 0.293$), as well as significant Group × Time ($p < 0.001$; $\eta^2 = 0.241$), Group × Cast ($p = 0.0241$; $\eta^2 = 0.062$), Time × Cast ($p < 0.001$; $\eta^2 = 0.321$), and Group × Time × Cast interactions ($p < 0.001$; $\eta^2 = 0.210$). Percent difference between pre- and post-cast passive tension (Supplemental Figure 2b) showed a significant main effect of Time ($p = 0.00221$; $\eta^2 = 0.361$), but no significant Group × Time interaction ($p = 0.449$; $\eta^2 = 0.063$), indicating that the magnitude of cast-induced increases in passive tension evolved similarly across recovery in intact and OVX groups. Immediately following cast-removal, passive tension was significantly increased compared to pre-cast values in both groups (+242% and +283% from pre-cast, respectively $p < 0.001$). Across recovery, passive tension progressively decreased such that by week 2, both groups no longer significantly differed from pre-cast values (+86% and +84% from pre-cast, respectively $p > 0.05$). In the intact group, however, passive tension at the week 4 timepoint was significantly higher than baseline (+136% from pre-cast, $p = 0.00821$).

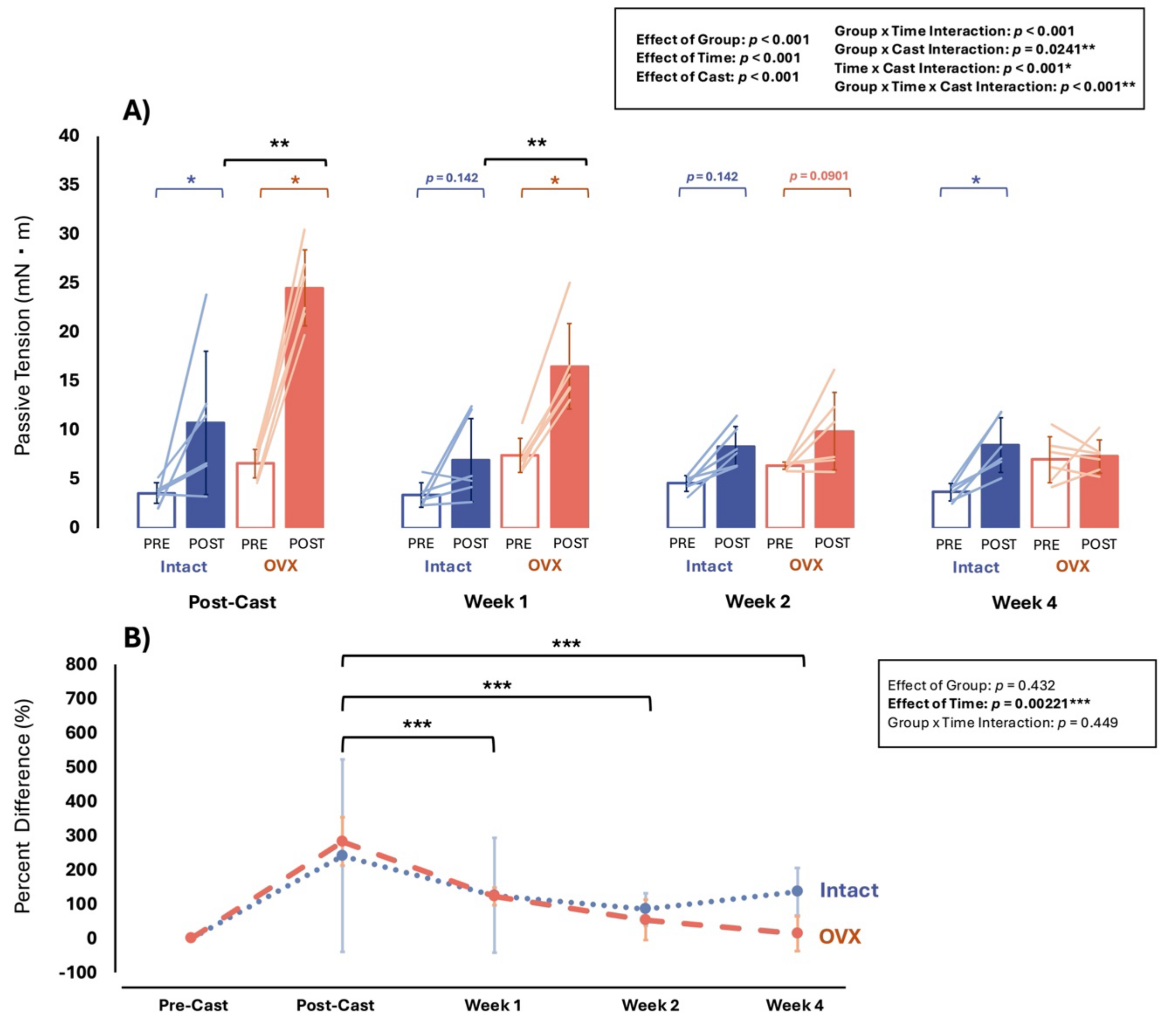

***Supplemental Figure 2*: Differences in plantar-flexor passive tension at a 90° ankle angle** in intact (n = 24) and ovariectomized (OVX; n = 24) adult female rats across recovery timepoints following unilateral hindlimb immobilization in a shortened position. **(A) Absolute pre- and post-cast torque values** for intact and OVX rats at post-cast, 1 week, 2 weeks, and 4 weeks of recovery (n = 6/group per timepoint). Horizontal lines represent the decrease in pre- to post- cast passive tension for individual rats. Statistical comparisons for Panel A were performed using a three-way ANOVA (Group × Time × Cast). **(B) Percent difference in passive tension from pre-cast to post-cast** for each rat across the same recovery timepoints. Statistical comparisons for Panel B were performed using a two-way ANOVA (Group × Time) on percent-difference values. Data are displayed as mean ± standard deviation. *Significant pre–post difference within a group at that timepoint (Panel A; $p < 0.05$). **Significant difference between intact and OVX post-cast values at that timepoint (Panel A; $p < 0.05$). ***Significant difference from the post-cast timepoint when pre- and post-cast values are combined, with black bars representing groups combined (Panel B; $p < 0.05$).

3. Passive Tension at a 70 Degree Ankle Angle

Absolute passive tension at a 70° ankle angle (Supplemental Figure 3a) showed significant effects of Group ($p < 0.001$; $\eta^2 = 0.182$), Time ($p < 0.001$; $\eta^2 = 0.218$), and Cast ($p < 0.001$; $\eta^2 = 0.664$), as well as significant Group × Time ($p = 0.0373$; $\eta^2 = 0.100$) and Time × Cast interactions ($p < 0.001$; $\eta^2 = 0.261$), with a trending, but nonsignificant Group × Time × Cast interaction ($p = 0.0603$; $\eta^2 = 0.088$). Percent difference between pre- and post-cast passive tension (Supplemental Figure 3b) showed significant effects of Group ($p = 0.0351$; $\eta^2 = 0.107$) and Time ($p = 0.00322$; $\eta^2 = 0.287$), but no Group × Time interaction ($p = 0.585$; $\eta^2 = 0.047$), indicating that intact and OVX groups differed in overall magnitude of passive tension increases but had similar recovery trajectories. Immediately following cast-removal, passive tension was significantly increased in both groups (+335% and +304% from pre-cast, respectively $p < 0.001$). Across recovery, passive tension progressively decreased in both groups but remained significantly higher than pre-cast values across all timepoints ($p < 0.05$), with a greater magnitude of passive tension increase in the intact group overall ($p = 0.0351$).

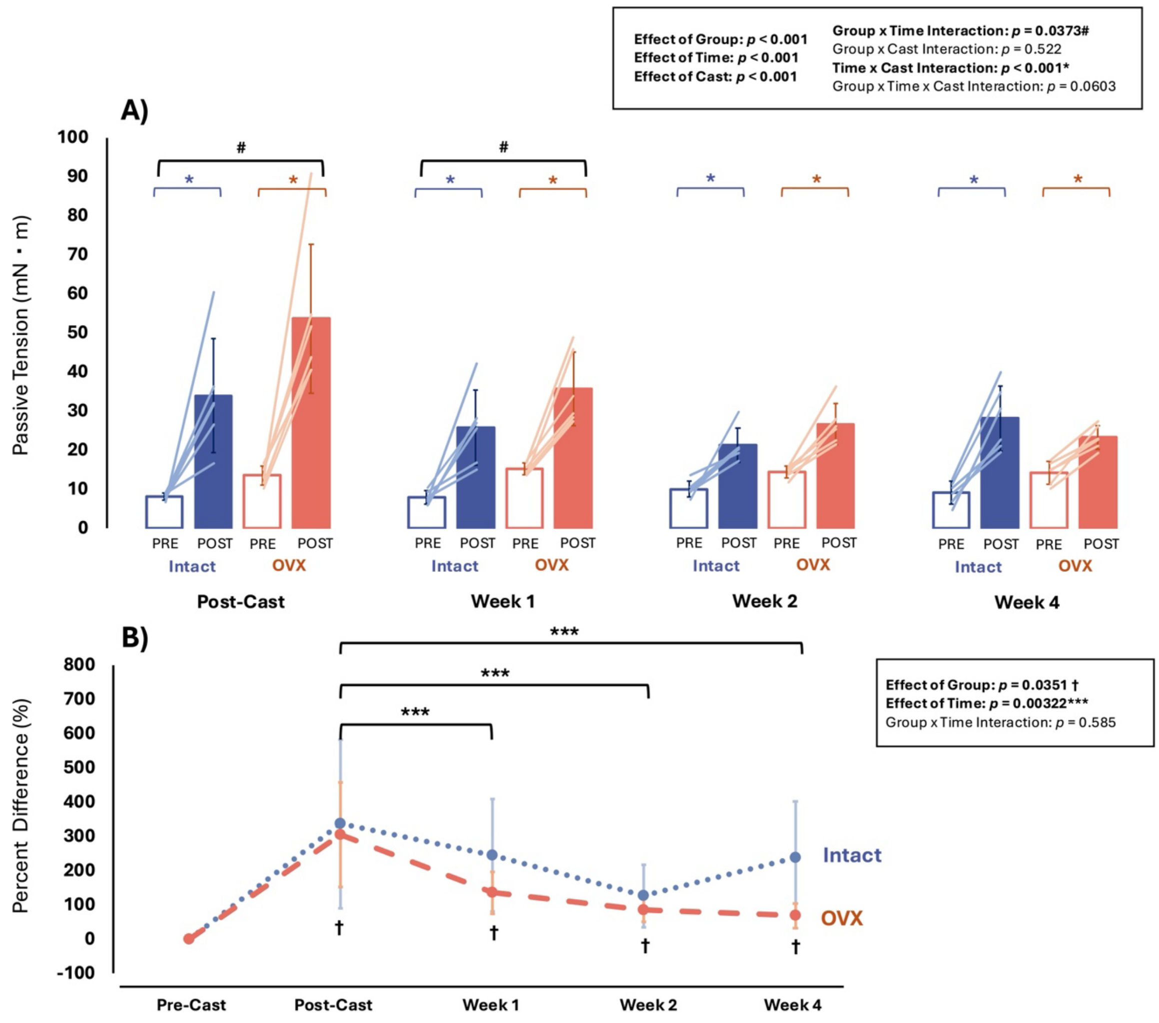

Supplemental Figure 3**: Differences in plantar-flexor passive tension at a 70° ankle angle** in intact (n = 24) and ovariectomized (OVX; n = 24) adult female rats across recovery timepoints following unilateral hindlimb immobilization in a shortened position. **(A) Absolute pre- and post-cast torque values** for intact and OVX rats at post-cast, 1 week, 2 weeks, and 4 weeks of recovery (n = 6/group per timepoint). Horizontal lines represent the decrease in pre- to post- cast passive tension for individual rats. Statistical comparisons for Panel A were performed using a three-way ANOVA (Group × Time × Cast). **(B) Percent difference in passive tension from pre-cast to post-cast** for each rat across the same recovery timepoints. Statistical comparisons for Panel B were performed using a two-way ANOVA (Group × Time) on percent-difference values. Data are displayed as mean ± standard deviation. *Significant pre–post difference within a group at that timepoint (Panel A; $p < 0.05$). #Significant difference between intact and OVX groups at that timepoint with pre and post values combined (Panel A; $p < 0.05$). † Significant difference between intact and OVX groups percent difference across all timepoints (Panel B; $p < 0.05$). ***Significant difference from the post-cast timepoint when pre- and post-cast values are combined, with black bars representing groups combined (Panel B; $p < 0.05$).

4. Maximum Shortening Velocity

Absolute maximum plantar-flexor shortening velocity (Supplemental Figure 4a) showed significant effects of Group ($p < 0.001$; $\eta^2 = 0.485$) and Cast ($p < 0.001$; $\eta^2 = 0.571$), as well as significant Group × Time ($p < 0.001$; $\eta^2 = 0.230$), Group × Cast ($p < 0.001$; $\eta^2 = 0.295$), and Time x Cast interactions ($p < 0.001$; $\eta^2 = 0.246$), but no significant Group x Time x Cast interaction ($p = 0.407$; $\eta^2 = 0.035$). Across all recovery timepoints, the OVX group exhibited substantially lower pre-cast maximum velocities compared to the intact group ($p < 0.001$), indicating baseline group differences independent of immobilization. Percent difference between pre- and post-cast maximum velocity (Supplemental Figure 4b) showed significant effects of Group ($p < 0.001$; $\eta^2 = 0.310$) and Time ($p < 0.001$; $\eta^2 = 0.475$), with a significant Group × Time interaction ($p = 0.0211$; $\eta^2 = 0.214$), indicating that the recovery of cast-induced velocity deficits differed between intact and OVX groups. Immediately following cast-removal, both intact and OVX groups exhibited significant reductions in maximum velocity (-54% and -51% from pre-cast, respectively $p < 0.001$). In the intact group, percent-difference values at week 1 did not differ significantly from the post-cast timepoint (-43% vs -54% from pre-cast, $p > 0.05$), indicating limited early recovery of maximum velocity. In contrast, the OVX group showed significant differences from the post-cast timepoint at all recovery weeks ($p < 0.05$). Direct comparisons between intact and OVX groups revealed significant differences in percent difference at week 1 (-43% and -6% from pre-cast, respectively $p < 0.001$) and week 4 (-25% and +6% from pre-cast, respectively $p < 0.001$). Despite these differences in relative change, absolute maximum velocity in the OVX group remained consistently lower than that of the intact group across all timepoints except week 4 ($p < 0.001$).

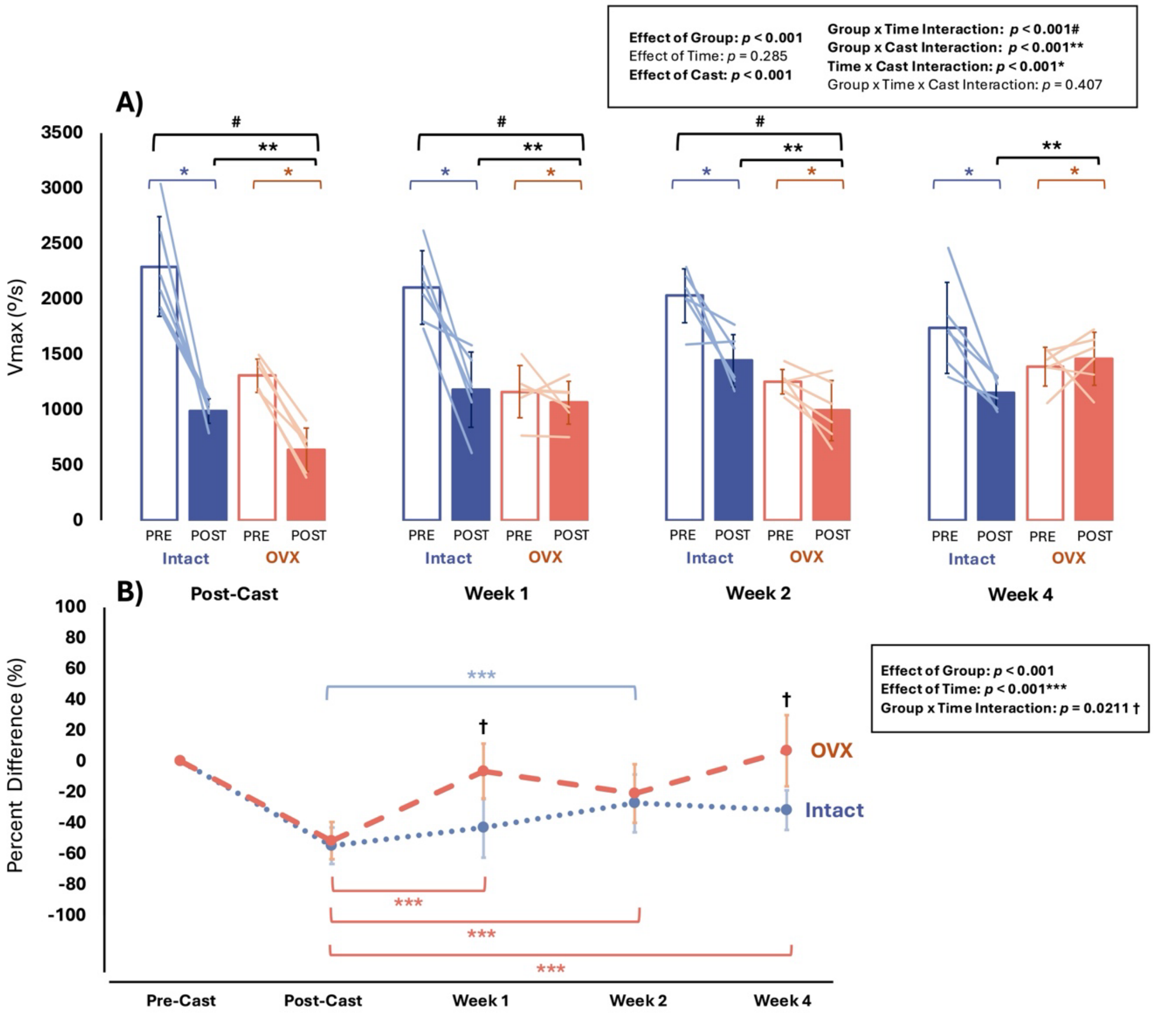

***Supplemental Figure 4*: Differences in maximum plantar-flexor velocity** in intact (n = 24) and ovariectomized (OVX; n = 24) adult female rats across recovery timepoints following unilateral hindlimb immobilization in a shortened position. **(A) Absolute pre- and post-cast torque values** for intact and OVX rats at post-cast, 1 week, 2 weeks, and 4 weeks of recovery (n = 6/group per timepoint). Horizontal lines represent the decrease in pre- to post- cast maximum velocity for individual rats. Statistical comparisons for Panel A were performed using a three-way ANOVA (Group × Time × Cast). **(B) Percent difference in maximum velocity from pre-cast to post-cast** for each rat across the same recovery timepoints. Statistical comparisons for Panel B were performed using a two-way ANOVA (Group × Time) on percent-difference values. Data are displayed as mean ± standard deviation. *Significant pre–post difference with groups combined across time (Panel A; $p < 0.05$). **Significant difference between intact and OVX post-cast values with timepoints combined (Panel A; $p < 0.05$). #Significant difference between intact and OVX groups at that timepoint with pre and post values combined (Panel A; $p < 0.05$). † Significant difference between intact and OVX groups percent difference at that timepoint (Panel B; $p < 0.05$). ***Significant difference from the post-cast timepoint when pre- and post-cast values are combined: purple-coloured bars represent intact group, orange-coloured bars represent OVX group (Panel B; $p < 0.05$).

5. Velocity at Peak Power

Absolute velocity at peak power (optimal velocity; Supplemental Figure 5a) showed significant effects of Group ($p < 0.001$; $\eta^2 = 0.510$), Time ($p < 0.001$; $\eta^2 = 0.196$), and Cast ($p < 0.001$; $\eta^2 = 0.686$), as well as significant Group × Time ($p < 0.001$; $\eta^2 = 0.244$), Group × Cast ($p < 0.001$; $\eta^2 = 0.266$), and Time × Cast interactions ($p < 0.001$; $\eta^2 = 0.303$), but no significant Group x Time x Cast interaction ($p = 0.223$; $\eta^2 = 0.053$). Across all recovery timepoints, the OVX group exhibited substantially lower pre-cast optimal velocities compared to the intact group ($p < 0.001$), indicating large baseline group differences independent of immobilization. Percent difference between pre- and post-cast optimal velocity (Supplemental Figure 5b) showed significant effects of Group ($p < 0.001$; $\eta^2 = 0.255$) and Time ($p < 0.001$; $\eta^2 = 0.548$), with a significant Group × Time interaction ($p = 0.0382$; $\eta^2 = 0.188$), indicating that both the magnitude and recovery of cast-induced optimal velocity deficits differed between intact and OVX groups. Immediately following cast-removal, both intact and OVX groups exhibited significant reductions in optimal velocity (-50% and -48% from pre-cast, respectively $p < 0.001$). In the intact group, percent-difference values at week 1 did not differ significantly from the post-cast timepoint (-39% from pre-cast, $p = 0.324$), indicating limited early recovery of optimal velocity. In contrast, the OVX group showed significant differences from the post-cast timepoint at all recovery weeks ($p < 0.05$). Direct comparisons between intact and OVX groups revealed significant differences in percent difference at week 1 (-39% and -10% from pre-cast, respectively $p < 0.001$) and week 4 (-25% and -6% from pre-cast, respectively $p = 0.0122$). Despite these differences in relative change, absolute optimal velocity values in the OVX group remained consistently lower than that of the intact group across all timepoints except week 4.

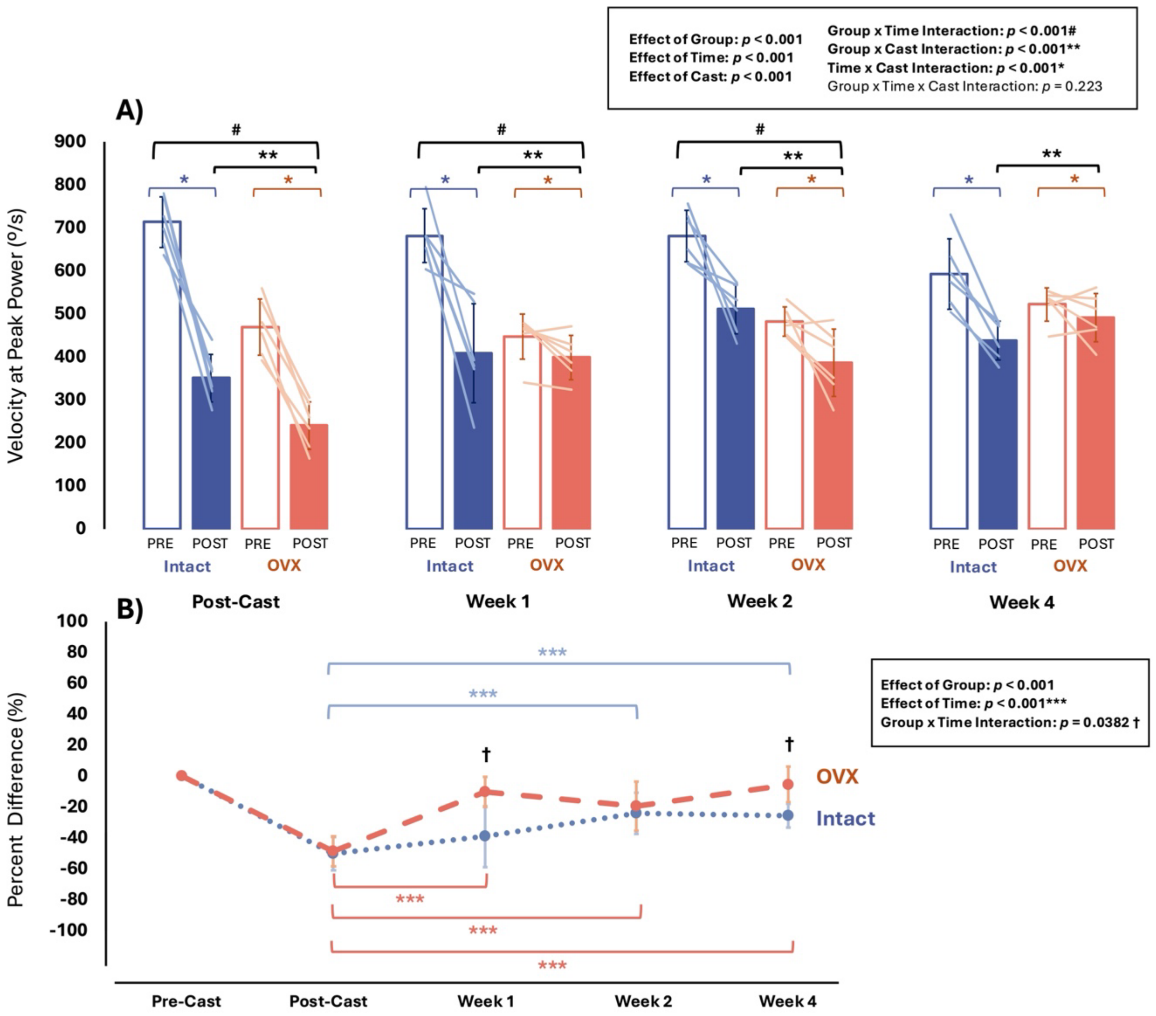

***Supplemental Figure 5*: Differences in optimal plantar-flexor velocity** in intact (n = 24) and ovariectomized (OVX; n = 24) adult female rats across recovery timepoints following unilateral hindlimb immobilization in a shortened position. **(A) Absolute pre- and post-cast torque values** for intact and OVX rats at post-cast, 1 week, 2 weeks, and 4 weeks of recovery (n = 6/group per timepoint). Horizontal lines represent the decrease in pre- to post- cast optimal velocity for individual rats. Statistical comparisons for Panel A were performed using a three-way ANOVA (Group × Time × Cast). **(B) Percent difference in optimal velocity from pre-cast to post-cast** for each rat across the same recovery timepoints. Statistical comparisons for Panel B were performed using a two-way ANOVA (Group × Time) on percent-difference values. Data are displayed as mean ± standard deviation. *Significant pre–post difference with groups combined across time (Panel A; $p < 0.05$). **Significant difference between intact and OVX post-cast values with timepoints combined (Panel A; $p < 0.05$). #Significant difference between intact and OVX groups at that timepoint with pre and post values combined (Panel A; $p < 0.05$). † Significant difference between intact and OVX groups percent difference at that timepoint (Panel B; $p < 0.05$). ***Significant difference from the post-cast timepoint when pre- and post-cast values are combined: purple-coloured bars represent intact group, orange-coloured bars represent OVX group (Panel B; $p < 0.05$).

6. Torque at Peak Power

Absolute torque at peak power (optimal torque; Supplemental Figure 6a) showed significant effects of Time ($p < 0.001$; $\eta^2 = 0.251$) and Cast ($p < 0.001$; $\eta^2 = 0.397$), as well as significant Group x Time ($p = 0.0462$; $\eta^2 = 0.095$), Group × Cast ($p < 0.001$; $\eta^2 = 0.180$), Time × Cast ($p < 0.001$; $\eta^2 = 0.330$), and Group × Time × Cast interactions ($p < 0.001$; $\eta^2 = 0.243$). Percent difference between pre and post-cast optimal torque (Supplemental Figure 6b) showed significant effects of Time ($p < 0.001$; $\eta^2 = 0.670$) and Group ($p < 0.001$; $\eta^2 = 0.438$), and a significant Group x Time interaction ($p < 0.001$; $\eta^2 = 0.571$), indicating that both the magnitude and recovery of cast-induced optimal torque loss differed between intact and OVX groups. Immediately following cast-removal, intact and OVX groups experienced similarly large decreases in optimal torque (-33% and -27% from pre-cast respectively, $p < 0.001$). However, the recovery trajectory differed between groups. In the intact group, optimal torque increased steadily across the recovery period and by week 2 no longer differed from pre-cast values (+4% from pre-cast, $p = 0.533$), ultimately surpassing baseline by week 4 (+40% from pre-cast, $p < 0.001$). In contrast, the OVX group remained significantly impaired at week 2 (–27% from pre-cast, $p < 0.001$) and continued to show a persistent torque deficit at week 4 (–21% from pre-cast, $p = 0.00211$).

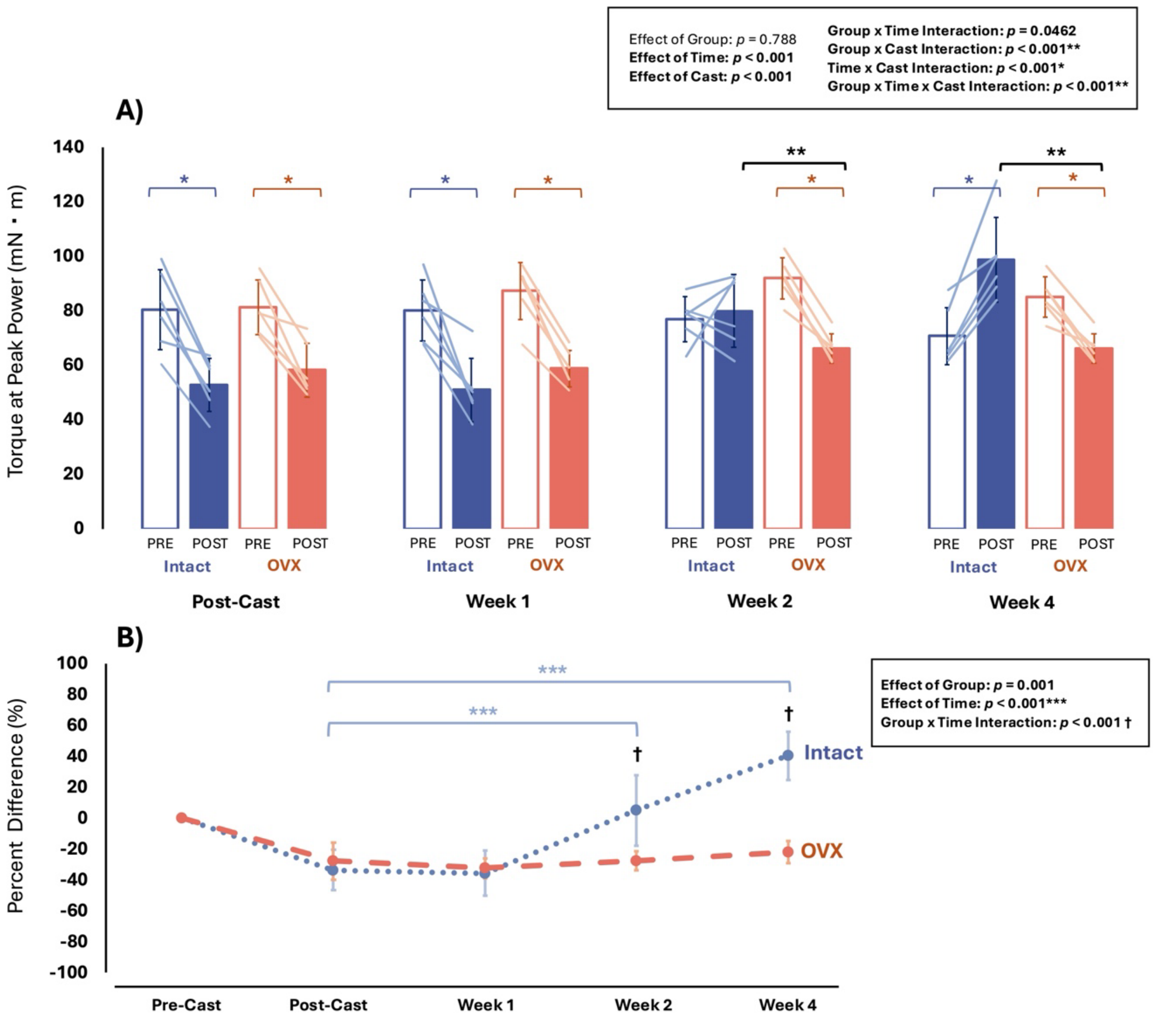

***Supplemental Figure 6*: Differences in optimal plantar-flexor torque** in intact (n = 24) and ovariectomized (OVX; n = 24) adult female rats across recovery timepoints following unilateral hindlimb immobilization in a shortened position. **(A) Absolute pre- and post-cast torque values** for intact and OVX rats at post-cast, 1 week, 2 weeks, and 4 weeks of recovery (n = 6/group per timepoint). Horizontal lines represent the decrease in pre- to post- cast optimal torque values for individual rats. Statistical comparisons for Panel A were performed using a three-way ANOVA (Group × Time × Cast). **(B) Percent difference in optimal torque from pre-cast to post-cast** for each rat across the same recovery timepoints. Statistical comparisons for Panel B were performed using a two-way ANOVA (Group × Time) on percent-difference values. Data are displayed as mean ± standard deviation. *Significant pre–post difference within a group at that timepoint (Panel A; $p < 0.05$). **Significant difference between intact and OVX post-cast values at that timepoint (Panel A; $p < 0.05$). † Significant difference between intact and OVX groups percent difference at that timepoint (Panel B; $p < 0.05$). ***Significant difference from the post-cast timepoint when pre- and post-cast values are combined: purple-coloured bars represent intact group, orange-coloured bars represent OVX group (Panel B; $p < 0.05$).

7. Soleus Fascicle Length

Absolute soleus FL (Supplemental Figure 8a) showed significant effects of Group ($p < 0.001$; $\eta^2 = 0.486$), Time ($p < 0.001$; $\eta^2 = 0.174$), and Cast ($p < 0.001$; $\eta^2 = 0.204$), as well as a significant Time × Cast interaction ($p = 0.0252$; $\eta^2 = 0.110$), but no significant Group × Time × Cast interaction ($p = 0.771$; $\eta^2 = 0.014$). The OVX group exhibited shorter soleus FL than the intact group when legs were combined across time, indicating a main effect of Group. Percent difference between CON and CAST soleus FL (Supplemental Figure 8b) showed significant effects of Group ($p < 0.001$; $\eta^2 = 0.227$) and Time ($p < 0.001$; $\eta^2 = 0.377$), but no significant Group × Time interaction ($p = 0.442$; $\eta^2 = 0.064$), indicating that intact and OVX groups differed in overall magnitude of FL deficits but had similar recovery trajectories. Immediately following cast-removal, the OVX group experienced greater deficits in FL compared to the intact group (-15% and -11% from CON, respectively, $p < 0.001$). Across recovery, soleus FL progressively increased toward CON values, with significant differences from the post-cast timepoint observed at the week 2 ($p = 0.00332$) and week 4 ($p < 0.001$) recovery timepoints in both OVX and intact rats.

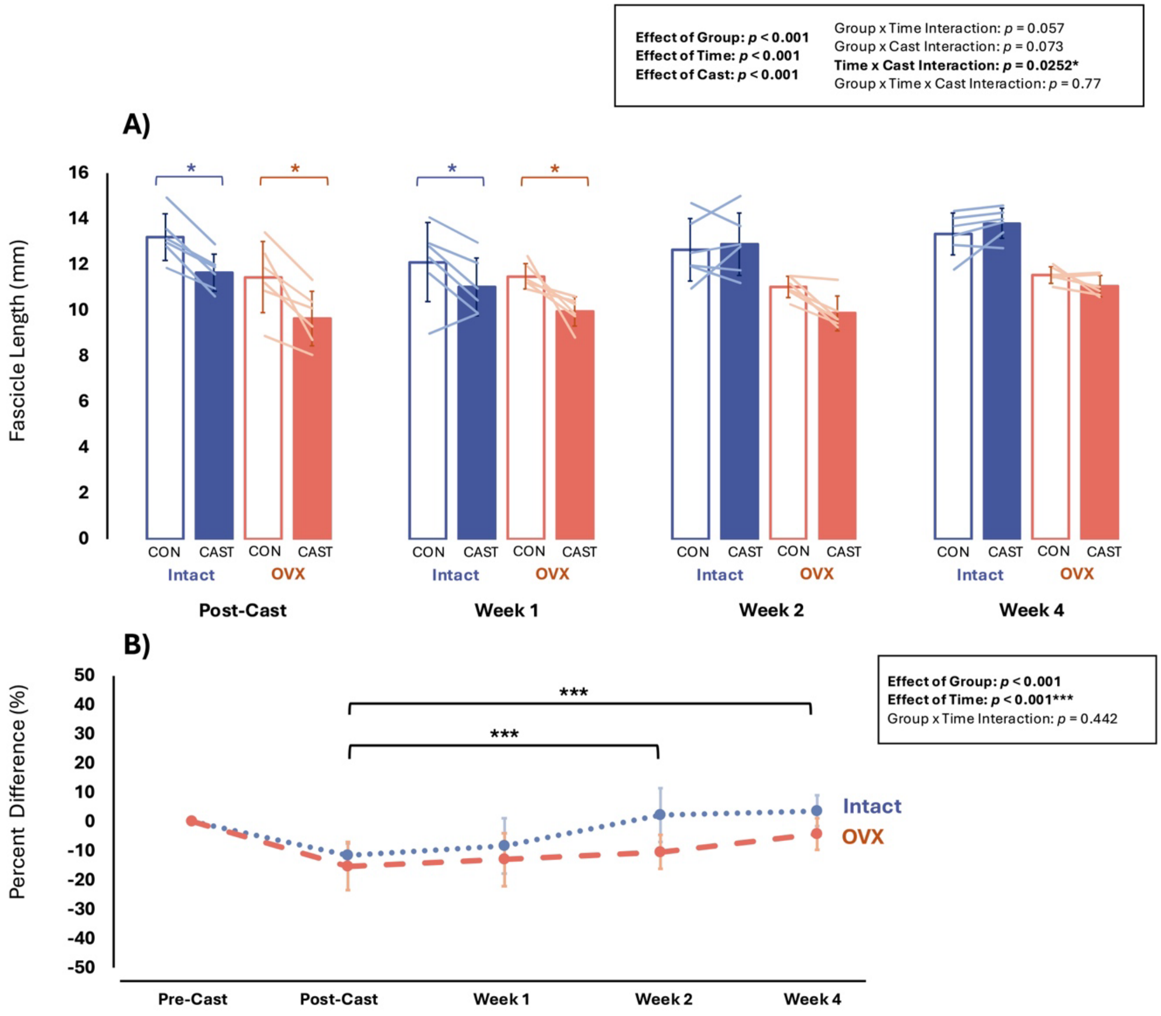

***Supplemental Figure 7*: Differences in soleus fascicle length (FL)** in intact (n = 24) and ovariectomized (OVX; n = 24) adult female rats following unilateral hindlimb immobilization in a shortened position. **(A) Absolute soleus FL values** for the control (CON) and casted (CAST) legs of intact and OVX rats at post-cast, 1 week, 2 weeks, and 4 weeks of recovery (n = 6/group per timepoint). Horizontal lines represent the difference in CON and CAST FL values for individual rats. Statistical comparisons for Panel A were performed using a three-way ANOVA (Group × Time × Cast). **(B) Percent difference in soleus FL** between CON and CAST legs for each rat across the same recovery timepoints. Statistical comparisons for Panel B were performed using a two-way ANOVA (Group × Time) on percent-difference values. Data are displayed as mean ± standard deviation. *Significant CON–CAST difference at specific timepoints with groups combined (Panel A; $p < 0.05$). † Significant main effect of group when legs are combined, averaged across time (Panel B; $p < 0.05$). *** Significant difference from the post-cast timepoint when CON and CAST values are combined, with black bars representing groups combined (Panel B; $p < 0.05$).

8. Soleus Sarcomere Length

Absolute soleus SL (Supplemental Figure 9a) showed a significant effect of Group ($p < 0.001$; $\eta^2 = 0.131$) and Cast ($p < 0.001$; $\eta^2 = 0.661$), but no significant interactions. Percent difference between CON and CAST soleus SL (Supplemental Figure 9b) likewise showed no significant effects of Group ($p = 0.170$; $\eta^2 = 0.046$), Time (p =0.262; 0.001; $\eta^2 = 0.094$), or a Group × Time interaction ($p = 0.972$; $\eta^2 = 0.006$), indicating that changes in soleus SL immediately following cast-removal were similar between intact and OVX groups (+6% and +8% from CON, respectively $p < 0.05$) and remained relatively stable across recovery.

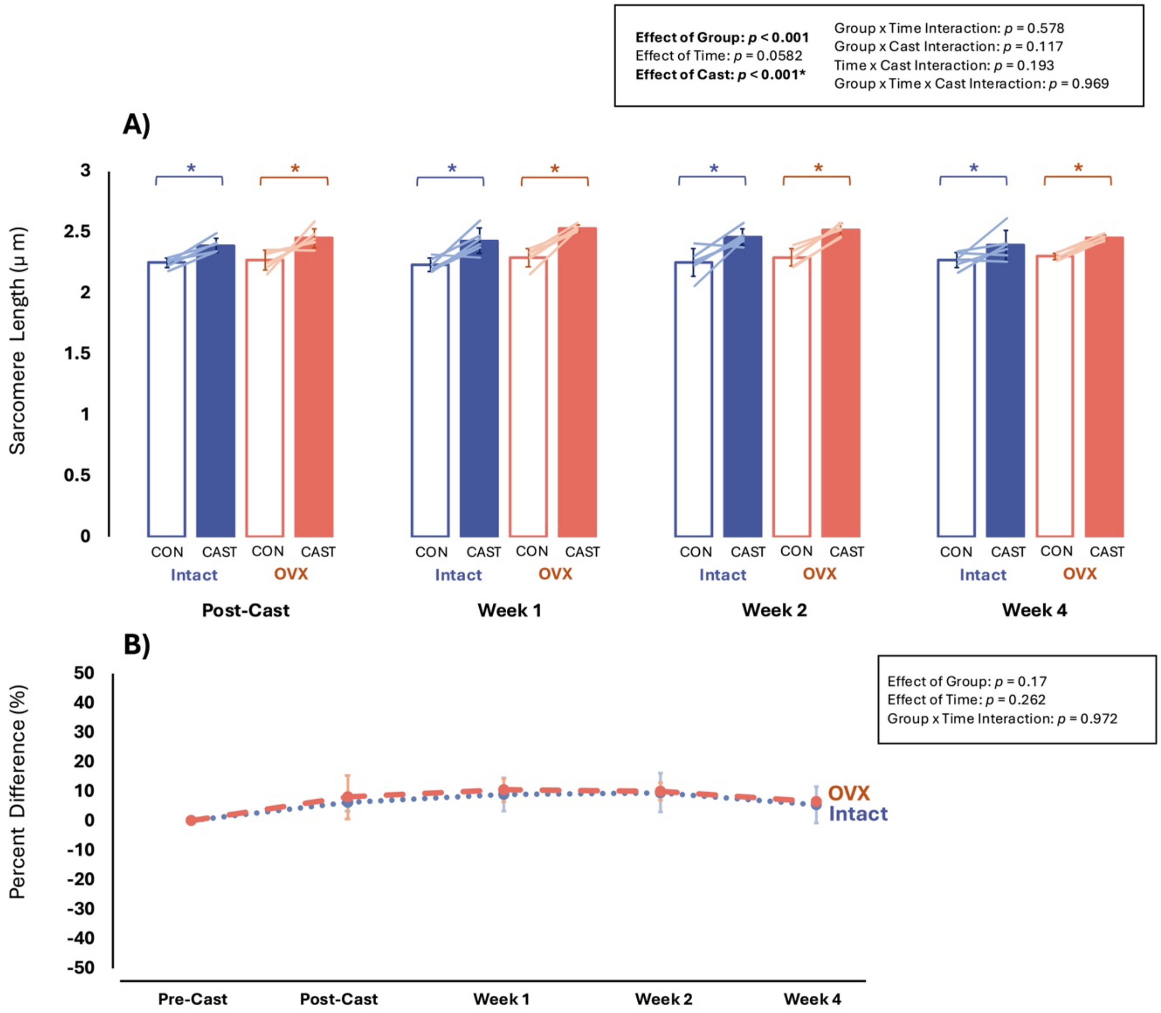

***Supplemental Figure 8*: Differences in soleus sarcomere length (SL)** in intact (n = 24) and ovariectomized (OVX; n = 24) adult female rats following unilateral hindlimb immobilization in a shortened position. **(A) Absolute soleus SL values** for the control (CON) and casted (CAST) legs of intact and OVX rats at post-cast, 1 week, 2 weeks, and 4 weeks of recovery (n = 6/group per timepoint). Horizontal lines represent the difference in CON and CAST SL values for individual rats. Statistical comparisons for Panel A were performed using a three-way ANOVA (Group × Time × Cast). **(B) Percent difference in soleus SL** between CON and CAST legs for each rat across the same recovery timepoints. Statistical comparisons for Panel B were performed using a two-way ANOVA (Group × Time) on percent-difference values. Data are displayed as mean ± standard deviation. *Significant CON–CAST difference averaged across time with groups combined (Panel A; $p < 0.05$).

9 Medial Gastrocnemius Fascicle Length

Absolute MG FL (Supplemental Figure 10a) showed significant effects of Group ($p < 0.001$; $\eta^2 = 0.532$), Time ($p = 0.00332$; $\eta^2 = 0.157$), and Cast ($p < 0.001$; $\eta^2 = 0.189$), as well as a significant Time × Cast interaction ($p < 0.001$; $\eta^2 = 0.194$), but no significant Group × Time × Cast interaction ($p = 0.496$; $\eta^2 = 0.029$). The OVX group exhibited shorter MG FL than the intact group when legs were combined across time, indicating a main effect of Group. Percent difference between CON and CAST MG FL (Supplemental Figure 10b) showed significant effects of Group ($p = 0.0155$; $\eta^2 = 0.138$) and Time ($p < 0.001$; $\eta^2 = 0.455$), but no significant Group × Time interaction ($p = 0.281$; $\eta^2 = 0.090$), indicating that intact and OVX groups differed in overall magnitude of FL deficits but had similar recovery trajectories. Immediately following cast-removal, intact and OVX groups experienced similar deficits in MG FL (-14% and -13% from CON, respectively $p < 0.001$). Across recovery, MG FL progressively increased toward CON values, with significant differences from the post-cast timepoint observed at the week 2 and week 4 recovery timepoints when groups were combined ($p < 0.001$) and trending significance at the week 1 timepoint ($p = 0.0571$).

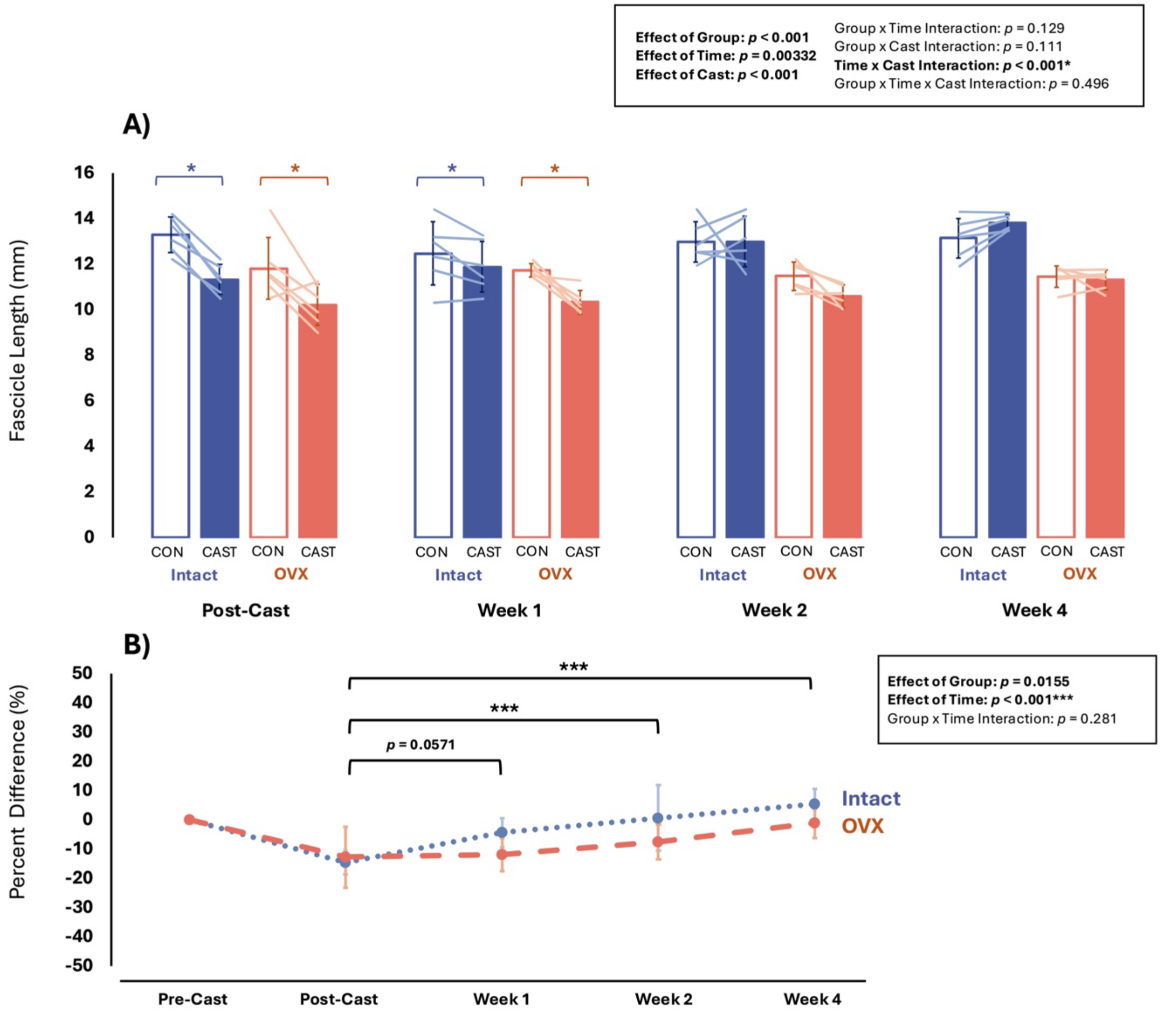

Supplemental Figure 9: **Differences in medial gastrocnemius (MG) fascicle length (FL)** in intact (n = 24) and ovariectomized (OVX; n = 24) adult female rats following unilateral hindlimb immobilization in a shortened position. **(A) Absolute MG FL values** for the control (CON) and casted (CAST) legs of intact and OVX rats at post-cast, 1 week, 2 weeks, and 4 weeks of recovery (n = 6/group per timepoint). Horizontal lines represent the difference in CON and CAST FL values for individual rats. Statistical comparisons for Panel A were performed using a three-way ANOVA (Group × Time × Cast). **(B) Percent difference in MG FL** between CON and CAST legs for each rat across the same recovery timepoints. Statistical comparisons for Panel B were performed using a two-way ANOVA (Group × Time) on percent-difference values. Data are displayed as mean ± standard deviation. *Significant CON–CAST difference at specific timepoints with groups combined (Panel A; $p < 0.05$). † Significant main effect of group when legs are combined, averaged across time (Panel B; $p < 0.05$). *** Significant difference from the post-cast timepoint when CON and CAST values are combined, with black bars representing groups combined (Panel B; $p < 0.05$).

10. Medial Gastrocnemius Sarcomere Length

Absolute MG SL (Supplemental Figure 11a) showed a significant effect of Group ($p < 0.001$; $\eta^2 = 0.304$), Time ($p = 0.0491$; $\eta^2 = 0.093$), and Cast ($p < 0.001$; $\eta^2 = 0.512$), with significant Group × Time ($p = 0.00552$; $\eta^2 = 0.147$) and Group × Cast ($p = 0.00552$; $\eta^2 = 0.096$) interactions, but no significant Group × Time × Cast interaction ($p = 0.662$; $\eta^2 = 0.020$). Across all timepoints, the OVX group exhibited substantially higher CON MG SL compared to the intact group ($p = 0.0372$), indicating large baseline group differences independent of immobilization. Percent difference between CON and CAST MG SL (Supplemental Figure 11b) showed a significant effect of Group ($p = 0.00444$; $\eta^2 = 0.185$) but no significant effect of Time ($p = 0.128$; $\eta^2 = 0.131$), or a significant Group × Time interaction ($p = 0.481$; $\eta^2 = 0.059$), indicating that changes in MG SL immediately following cast-removal were similar between intact and OVX groups (+2% and +8% from CON, respectively $p < 0.05$) and remained relatively stable across recovery, though the OVX group had higher overall MG SL increases from CON to CAST legs averaged across all timepoints ($p = 0.00421$).

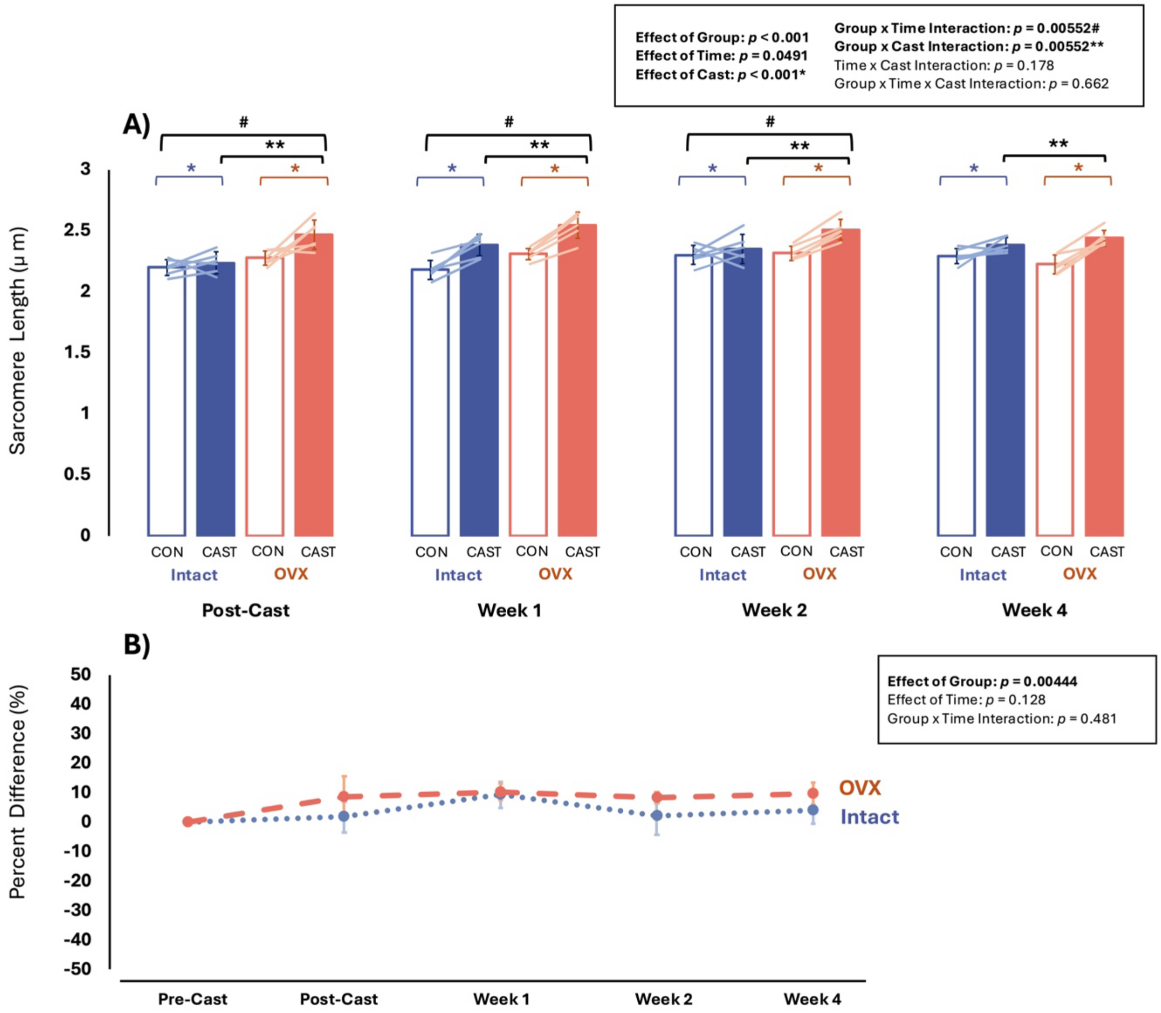

Supplemental Figure 10**: Differences in medial gastrocnemius (MG) sarcomere length (SL)** in intact (n = 24) and ovariectomized (OVX; n = 24) adult female rats following unilateral hindlimb immobilization in a shortened position. **(A) Absolute MG SL values** for the control (CON) and casted (CAST) legs of intact and OVX rats at post-cast, 1 week, 2 weeks, and 4 weeks of recovery (n = 6/group per timepoint). Horizontal lines represent the difference in CON and CAST SL values for individual rats. Statistical comparisons for Panel A were performed using a three-way ANOVA (Group × Time × Cast). **(B) Percent difference in MG SL** between CON and CAST legs for each rat across the same recovery timepoints. Statistical comparisons for Panel B were performed using a two-way ANOVA (Group × Time) on percent-difference values. Data are displayed as mean ± standard deviation. *Significant CON–CAST difference averaged across time with groups combined (Panel A; $p < 0.05$). **Significant difference between intact and OVX CAST values averaged across time (Panel A; $p < 0.05$). #Significant difference between intact and OVX groups at that timepoint with CON and CAST values combined (Panel A; $p < 0.05$). † Significant main effect of group when legs are combined, averaged across time (Panel B; $p < 0.05$).

11. Body Mass

Body mass (Supplemental Figure 12a) demonstrated significant main effects of Group ($p < 0.001$; $\eta^2 = 0.490$), Time ($p < 0.001$; $\eta^2 = 0.445$), and a significant Time × Cast interaction ($p < 0.001$; $\eta^2 = 0.519$). Significant Group × Cast ($p = 0.0601$; $\eta^2 = 0.083$) and Group × Time × Cast interactions were not observed ($p = 0.226$; $\eta^2 = 0.100$). Percent difference in body mass between pre- and post-cast measurements (Supplemental Figure 12b) showed a significant effect of Time ($p < 0.001$; $\eta^2 = 0.587$) and a significant Group × Time interaction ($p = 0.00322$; $\eta^2 = 0.289$), while the main effect of Group was not significant ($p = 0.0602$; $\eta^2 = 0.085$), indicating that body mass recovery differed between intact and OVX animals across the recovery period. Immediately following cast removal, both intact and OVX groups demonstrated reductions in body mass relative to pre-cast values (–14.8% and –5.5%, respectively). The intact group remained significantly reduced at week 1 (–10.5% from pre-cast, $p = 0.287$), but recovered to values no longer different from pre-cast by week 2 (+2.4%, $p < 0.001$ vs post-cast) and further increased above baseline by week 4 (+9.7%, $p < 0.001$). In contrast, the OVX group showed smaller initial reductions in body mass following cast removal (–4.2% at week 1, $p = 0.0410$), with recovery delayed until week 4 (+14.9% from pre-cast, $p = 0.0191$). Between-group comparisons revealed that intact animals exhibited significantly greater body mass loss than OVX animals at both post-cast and week 1 ($p = 0.021$ and $p = 0.041$, respectively), whereas no group differences were observed at week 2 or week 4.

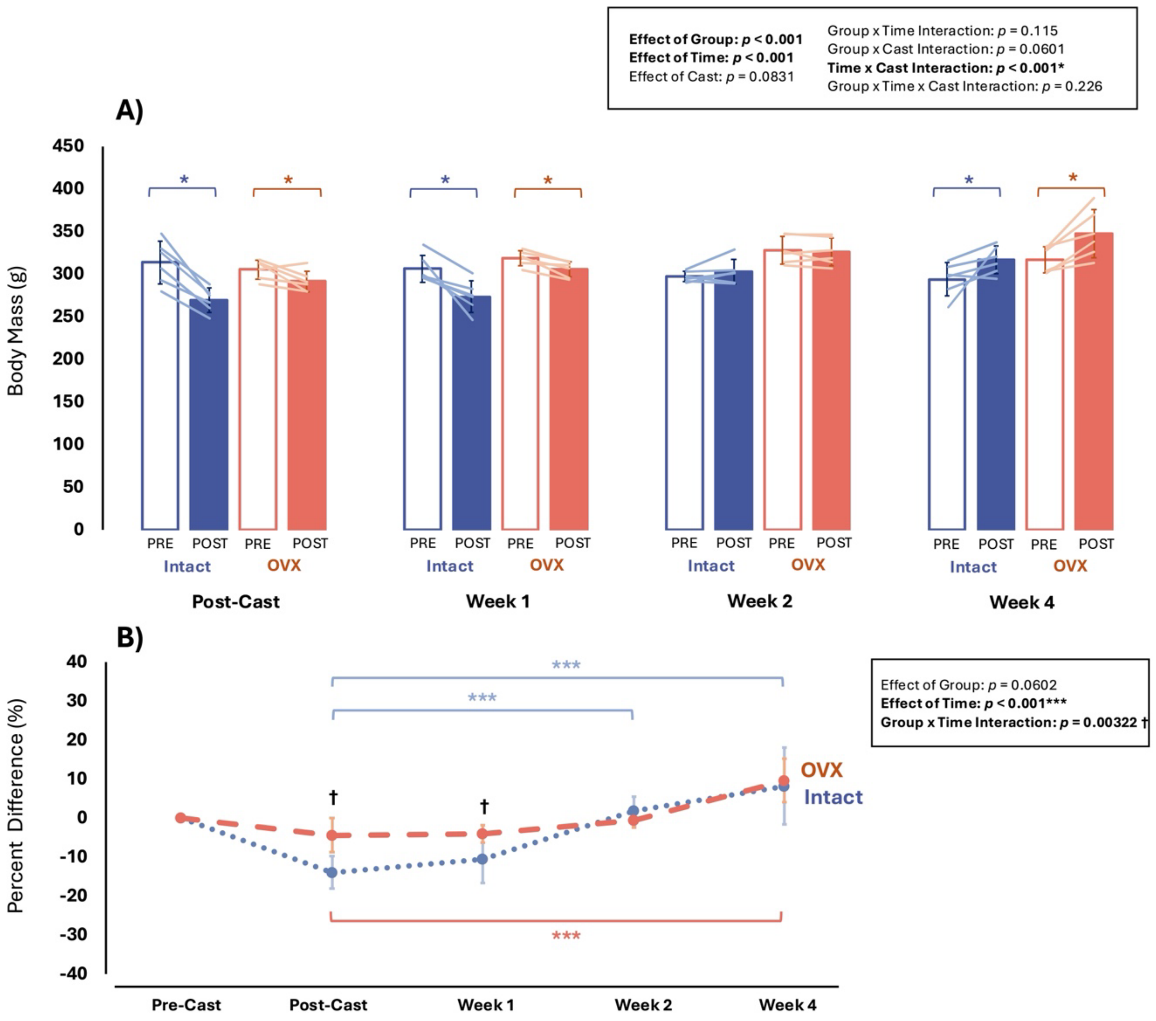

***Supplemental Figure 11*: Differences in body mass** in intact (n = 24) and ovariectomized (OVX; n = 24) adult female rats across recovery timepoints following unilateral hindlimb immobilization in a shortened position. **(A) Absolute pre- and post-cast body mass values** for intact and OVX rats at post-cast, 1 week, 2 weeks, and 4 weeks of recovery (n = 6/group per timepoint). Horizontal lines represent the decrease in pre- to post- cast optimal torque values for individual rats. Statistical comparisons for Panel A were performed using a three-way ANOVA (Group × Time × Cast). **(B) Percent difference in body mass from pre-cast to post-cast** for each rat across the same recovery timepoints. Statistical comparisons for Panel B were performed using a two-way ANOVA (Group × Time) on percent-difference values. Data are displayed as mean ± standard deviation. *Significant pre–post difference within a group at that timepoint (Panel A; $p < 0.05$). † Significant difference between intact and OVX groups percent difference at that timepoint (Panel B; $p < 0.05$). ***Significant difference from the post-cast timepoint when pre- and post-cast values are combined: purple-coloured bars represent intact group, orange-coloured bars represent OVX group (Panel B; $p < 0.05$).
